# Multifactor transcriptional control of alternative oxidase induction integrates diverse environmental inputs to enable fungal virulence

**DOI:** 10.1038/s41467-023-40209-w

**Published:** 2023-07-27

**Authors:** Zhongle Liu, Pauline Basso, Saif Hossain, Sean D. Liston, Nicole Robbins, Luke Whitesell, Suzanne M. Noble, Leah E. Cowen

**Affiliations:** 1 Department of Molecular Genetics, University of Toronto, Toronto, ON Canada; 2 UCSF Department of Microbiology & Immunology, San Francisco, CA USA; 3 UCSF Department of Medicine, Division of Infectious Diseases, San Francisco, CA USA

**Keywords:** Fungal genetics, Fungal immune evasion, Transcriptional regulatory elements

## Abstract

Metabolic flexibility enables fungi to invade challenging host environments. In *Candida albicans*, a common cause of life-threatening infections in humans, an important contributor to flexibility is alternative oxidase (Aox) activity. Dramatic induction of this activity occurs under respiratory-stress conditions, which impair the classical electron transport chain (ETC). Here, we show that deletion of the inducible *AOX2* gene cripples *C. albicans* virulence in mice by increasing immune recognition. To investigate further, we examined transcriptional regulation of *AOX2* in molecular detail under host-relevant, ETC-inhibitory conditions. We found that multiple transcription factors, including Rtg1/Rtg3, Cwt1/Zcf11, and Zcf2, bind and regulate the *AOX2* promoter, conferring thousand-fold levels of inducibility to *AOX2* in response to distinct environmental stressors. Further dissection of this complex promoter revealed how integration of stimuli ranging from reactive species of oxygen, nitrogen, and sulfur to reduced copper availability is achieved at the transcriptional level to regulate *AOX2* induction and enable pathogenesis.

## Abstract (web summary)

Metabolic flexibility allows fungi to invade hostile niches. Here, Liu et al. dissect the molecular mechanisms by which *Candida albicans* upregulates virulence-enabling alternative oxidase expression in response to host-relevant respiratory stresses.

## Introduction

Fungi have a profound, but underappreciated impact on human health, killing more than 1.5 million of the billions of people they infect worldwide each year^1–4^. Vulnerability to fungal infection is greatest for individuals with impaired immune function^5^. Of the deaths attributable to fungal infection in North America, ~90% are caused by species of *Candida*, *Aspergillus*, and *Cryptococcus*^6^. Among these, *Candida albicans* is one of the most frequent causes of invasive infection, with systemic candidiasis causing considerable morbidity and driving mortality rates as high as 40% despite treatment^7^. Poor outcome for invasive infections is attributable to the limited number of effective antifungals available and an alarming rise in frequency of clinical resistance.

The ability to utilize alternative carbon sources plays an essential role in allowing *C. albicans* to transition from a commensal colonizer to a pathogenic microbe^8–10^. During infection, this metabolic flexibility contributes to virulence and the emergence of drug resistance^11^. In particular, pathogenic fungi rely on respiration to utilize the poorly-fermentable carbon sources in host environments, not only for energy generation, but also to support essential anabolic processes^12,13^.

Under conditions compromising the classical electron transport chain (ETC), *C. albicans* makes use of a bypass respiratory pathway, which is supported by cyanide-insensitive alternative oxidases (Aox) located on the matrix side of the inner mitochondrial membrane^14,15^. Encoded by nuclear genes, constitutive (Aox1) and highly inducible (Aox2) isoforms catalyze electron transfer from ubiquinol to oxygen, without coupling it to proton-pumping. Found in diverse microbial pathogens, but not in humans, Aox is proposed as a potential drug target given its reported role in governing diverse cellular stress response pathways, including a role in maintaining cell wall architecture^16,17^. Early-stage studies with inhibitors, either alone or in combination with inhibitors of the ETC, have shown promise^16,18,19^. In *C. albicans* and other fungi, the importance of the classical ETC for virulence is well established^16,20,21^. In contrast, how Aox activity is regulated in response to stressors encountered during infection and how induction contributes to virulence have remained enigmatic.

## Results

### Inducible Aox activity supports systemic virulence in mice

**Fig. 1:**
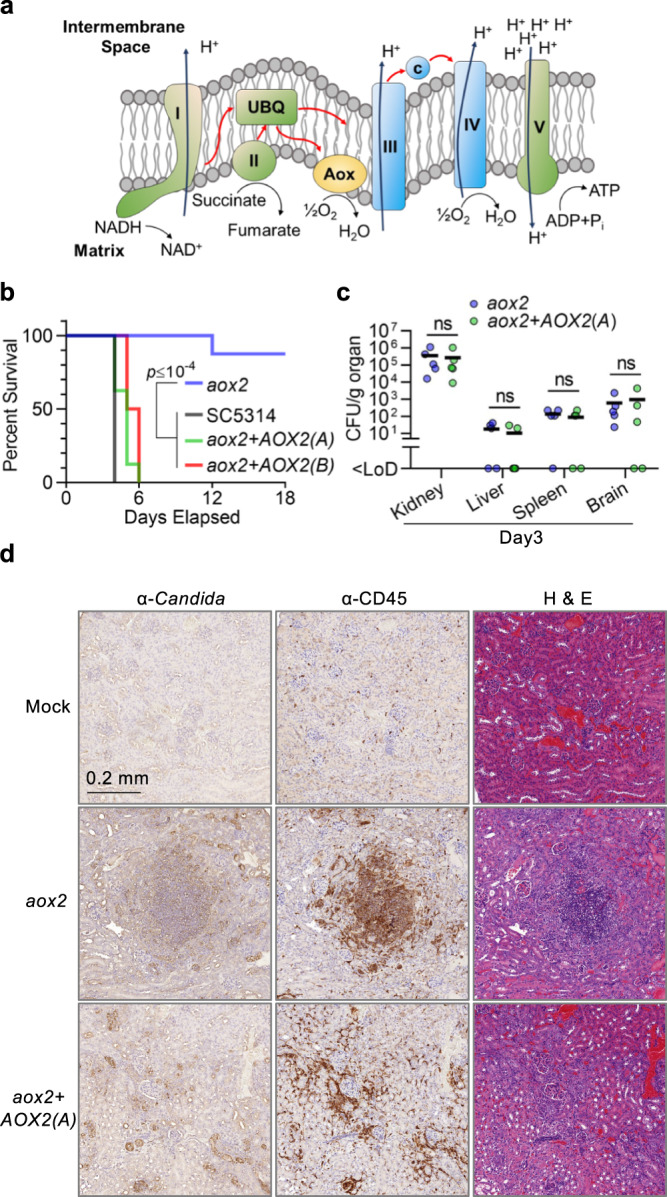
*AOX2* induction enables virulence in mice. **a** Schematic depiction of the mitochondrial electron transport chain (ETC) in *Candida* spp. The classical cytochrome pathway (COX; in blue) is composed of Complex III, cytochrome C, and Complex IV. Alternative oxidases (Aox) mediate direct electron transport from ubiquinol (UBQ) to molecular oxygen. Both COX and AOX pathways function with Complex I, Complex II, UBQ, and Complex V (components in green) to enable oxidative phosphorylation. **b** Alternative oxidase supports *C. albicans* virulence. Mice (*n* = 8) were infected with the indicated *C. albicans* strains by retro-orbital injection, and survival was monitored for 18 days. *p* values were calculated by Mantel–Cox test (*n* = 8): *aox2* vs. SC5314 (or *aox2* + *AOX2(B)*) *p* = 0.0001; *aox2* vs. *aox2* + *AOX2(A) p* < 0.0001). **c** An *aox2*-deletion strain is capable of initiating infection of target organs. Groups of mice (*n* = 5) were euthanized 3 days after systemic infection with *aox2* mutant or add-back strain (*aox2* + *AOX2(A)*), and fungal colony-forming units (CFUs) were recovered from the indicated organs. One kidney per animal was assessed for CFUs, and the other was used for the histological analyses presented in **d**; <LoD: less than limit of detection; ns: not significant (*p* > 0.05 by unpaired two-tailed *t* test for each organ). **d** An *aox2*-deletion mutant stimulates greater leukocyte accumulation at sites of infection than complemented add-back strain. Immunohistochemical staining of kidney sections (Day 3 post-infection) was performed using α-*Candida* or α-CD45 antibody (brown signals). H&E staining of a serial section is presented for orientation. Images are presented from representative tissue sections obtained from three mice for each experimental condition. Scale bar, 0.2 mm.

*Candida* spp. and other pathogenic microbes possess a bypass respiratory pathway supported by cyanide-insensitive alternative oxidases (Aox), which are located on the matrix side of the inner mitochondrial membrane (Fig. 1a). To learn whether Aox activity plays a role in the virulence of *C. albicans*, we focused primarily on *AOX2* due to its high level of inducibility in response to environmental challenges. We constructed an *AOX2* homozygous deletion mutant (referred to as *aox2*-deletion or *aox2*; the same nomenclature is used to reference all homozygous deletion mutants throughout this study). Upon systemic infection of immunocompetent mice, the *aox2*-deletion strain was significantly less virulent than the parental strain (Fig. 1b). Restoration of either allele of *AOX2*(A) or *AOX2*(B) was sufficient to restore virulence to a level equivalent to that of the wild-type strain (Fig. 1b).

To assess the proliferative capacity of the *aox2*-deletion strain within an infected host, we measured fungal burden in major organs following three days of systemic infection. The numbers of colony-forming units (CFU) recovered from *aox2*-infected kidneys, livers, spleens, and brains were comparable to those from tissues infected by the complemented strain (Fig. 1c). This result indicated that, although *AOX2* is required for progressive, lethal infection, it is dispensable for early events in pathogenesis. To determine whether differences in the host response contribute to the contrasting outcomes of infection with *aox2-deletion* vs. the complemented strain, we examined the extent of immune-cell accumulation in infected kidneys (organ of greatest fungal burden). Representative fixed sections of mock-infected, *aox2-*infected, and *aox2* + *AOX2(A)-*infected renal cortex are shown in Fig. 1d; as indicated in the figure panels, fungal cells were stained with anti-*Candida* antibody and immune cells with antibody against the leukocyte common antigen (LCA, also known as CD45). Throughout the renal cortex, invading *aox2* fungi induced markedly greater leukocyte accumulation than the complemented strain (Fig. 1d). Large dense foci of CD45^+^-cells were observed in non-contiguous sections of kidneys recovered from independent mice infected with the *aox2*-deletion strain. In contrast, relatively sparse, diffuse infiltration was observed in kidneys infected by the *aox2* + *AOX2(A)* strain (Fig. 1d; Supplementary Fig. 1a). Thus, greater leukocyte accumulation could be observed in the kidney during challenge with the *aox2* strain than with the complemented strain, even though overall fungal burdens were comparable at this very early stage of infection. Clearly, additional studies will be required in future work to determine the mechanisms underlying this differential accumulation, but plausible consequences could be enhanced host immune-mediated clearance of the *aox2* mutant at later time points and impaired virulence.

In addition to the evasion of host immune defenses, the ability to switch from yeast to filamentous morphologies is a key virulence trait of *C. albicans*. Periodic acid–Schiff (PAS) staining and α-*Candida* antibody IHC revealed numerous filamentous forms in *aox2-*infected kidneys (Supplementary Fig. 1b), indicating that the mutant retains the ability to undergo morphogenesis. This result is consistent with observations that *AOX2* is dispensable for filamentation under in vitro tissue culture conditions that mimic the host environment (Supplementary Fig. 1c).

In addition to its role as a fungal pathogen, *C. albicans* is primarily a normal component of the human gut microbiota. To determine whether *AOX2* is also required for commensal fitness, we tested the fitness of *aox2* in a mouse model of gut colonization^22^. *aox2-deletion* and complemented strains were introduced as a 1:1 mixed inoculum directly into the stomachs of the animal model, and the relative abundance of each strain in feces was monitored every five days for the next three weeks. As shown in Supplementary Fig. 1d, the *aox2* mutant maintained equivalent colonization to the complemented strain throughout the observed time-course, suggesting that *AOX2* is dispensable for fitness in the commensal niche.

### Aox functions in cooperation with the ETC to maintain alternative carbon source utilization

Previous studies have reported that *AOX2* expression is upregulated upon phagocytosis by murine macrophages^23^ and during progressive infection of kidneys in systemically infected mice^24^. Linking this evidence with our observations of a substantial virulence defect for *aox2* in a mouse model of systemic infection, we hypothesized that under host-relevant conditions, which compromise the classical ETC, *C. albicans* may increase Aox activity to maintain the respiratory metabolism that allows alternative carbon source utilization. To test this idea, we deleted both alleles of nuclear genes encoding subunits of each classical respiratory complex in wild-type and *aox1/aox2* double homozygous deletion (*aox*) backgrounds. Growth was compared on solid medium containing glucose or galactose, a poorly-fermentable sugar, as the primary carbon source. As a result of compromised oxidative phosphorylation, each deletion impaired growth on YP-galactose (YPGal) medium, with the most severe defect observed in the *atp2*-deletion strain. Combined with the deletion of *AOX* genes, however, growth of the cytochrome pathway mutants (*rip1*, *cyc1*, or *cox5*) was completely abolished on YPGal agar. These results suggest that, in strains that lack the cytochrome pathway, Aox activity supports sufficient respiration to utilize galactose. In contrast, the growth of strains lacking *NDH51* or *SDH12*, which retain an intact cytochrome pathway, was not further exacerbated on YPGal in the absence of Aox activity.

**Fig. 2:**
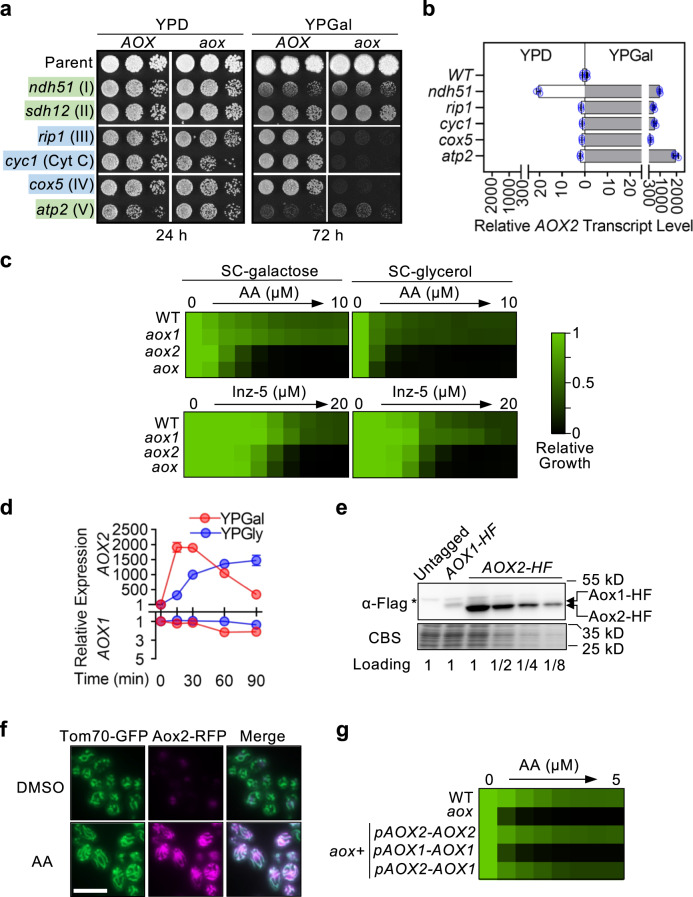
Induction of *AOX2* supports alternative carbon source utilization upon genetic or chemical compromise of classical respiration. **a** Aox activity is essential for *C. albicans* cytochrome pathway mutants to utilize galactose. Indicated genes were deleted in a wild-type (WT) or a homozygous *aox1/aox2*-deletion (*aox*) parent. Growth was monitored by spotting on YPD and YPGal agar. The respiratory complex impaired by each deletion is in parentheses. **b** Respiratory complex deletion mutants grown in galactose-containing medium overexpress *AOX2*. Expression in the wild-type (WT) was used to calculate relative *AOX2* levels across samples cultured in the same medium. **c** WT and mutant strains were grown in synthetic complete (SC) medium containing galactose or glycerol and two-fold serial dilutions of antimycin A (AA) or Inz-5. For each strain, growth (OD_600_) in drug-free medium was used to normalize growth in the presence of an inhibitor (see color bar). All dose-response assays are presented in the same format hereafter, unless otherwise specified. **d** Time-course of *AOX* induction upon AA treatment. *C. albicans* was treated with AA (10 μM) in YPGal or YPGly medium. *AOX1* and *AOX2* transcript levels were measured at each time point. Basal expression for each gene (time 0) was set to 1 for each condition. **e** Lysates were prepared from an untagged strain and strains expressing proteins tagged at the C-terminus with 6XHis-3XFlag (Aox1-HF or Aox2-HF) after 12-hours growth in YPGly+AA (10 μM), resolved by SDS-PAGE, and blots probed with anti-Flag antibody. Coomassie blue staining (CBS) of a replicate gel was used to confirm equal loading. The amount of Aox2-HF lysate was titered. Asterisk: the non-specific band. **f** Aox2-RFP fusion localizes to mitochondria. Images were acquired by fluorescence microscopy of cells that express Aox2-RFP (magenta) and Tom70-GFP (mitochondrial marker, green) following growth in YPGly containing AA (10 μM) or DMSO (0.1%). Scale bar, 10 μm. **g** *AOX1* functionally complements *AOX2* when driven by the inducible *AOX2* promoter. Relative growth inhibition by AA in SC-galactose medium is presented as in **c**. **b**, **d** present mean (SD) of technical triplicates from an experiment representative of biological duplicates with comparable results.

When cytochrome pathway mutants were grown in YPGal medium, *AOX2* levels were induced to several hundred-fold higher levels than that observed in the wild-type strain (Fig. 2b). Interestingly, *ndh51*- and *atp2*-deletion strains over-expressed *AOX2* in YPGal, to an even greater degree (Fig. 2b). Thus, *AOX2* induction seems to be a general response to compromise of oxidative phosphorylation, rather than a specific response to loss of cytochrome pathway activity. With glucose present as a carbon source (YPD), fermentative metabolism reduces dependence on respiration. Thus, in YPD, no ETC mutants required Aox activity for growth or induced *AOX2* to the same extent seen as observed in galactose medium (Fig. 2a, b).

### Chemical inhibition of the cytochrome pathway induces *AOX2* expression

By enabling temporal control, highly specific chemical inhibitors can complement genetic techniques in revealing how ETC function contributes to fungal metabolism in different environments. Antimycin A (AA), a classical Complex III inhibitor, reduced *C. albicans* growth in synthetic complete medium (SC) containing poorly fermentable (galactose) or non-fermentable (glycerol, lactate) carbon sources. The growth inhibition was strongly exacerbated by the deletion of both *AOX* genes (Fig. 2c; Supplementary Fig. 2a). Consistent with a previous report, we found that *AOX2* plays a dominant role in supporting alternative respiration^15^. Deletion of *AOX2* resulted in the same degree of hypersensitivity to AA as deletion of both paralogs, while deletion of *AOX1* alone did not alter sensitivity. Confirming the specificity of AA, dose-response assays using a structurally distinct, fungal-selective inhibitor of Complex III (Inz-5) yielded similar results in that deletion of *AOX2* increased sensitivity to the compound in poorly- or non-fermentable carbon sources (Fig. 2c; Supplementary Fig. 2a)^25,26^.

Use of AA allowed us to measure the kinetics of *AOX2* transactivation. In YPGal, *AOX2* transcripts increased sharply within 15 minutes upon addition of AA, while levels of *AOX1* remained constant (Fig. 2d). A decline in *AOX2* transcript level began after ~30 minutes post induction. *AOX2* was induced with different kinetics in cells utilizing glycerol (YPGly): expression increased gradually to a plateau with no decline seen over a 90-minute observation period (Fig. 2d). Compared with the response seen in YPGal or YPGly, induction in YPD was transient and low in magnitude (Supplementary Fig. 2b). Lack of strong, persistent induction in YPD likely reflects a reduced dependence on respiration under fermentation-enabling conditions. To test this hypothesis, we deleted *TYE7* and *GAL4*, which encode master transcriptional regulators of glycolytic gene expression in *C. albicans*^27^. This *tye7*/*gal4*-deletion strain was more dependent on respiration for energy production than its parental wild-type strain and was hypersensitive to inhibition of respiration in YPD (Supplementary Fig. 2c). Supporting our hypothesis, *AOX2* was strongly induced by AA in the *tye7*/*gal4*-deletion strain, even when utilizing glucose as a primary carbon source (Supplementary Fig. 2b). We conclude that *AOX2* inducibility by AA is tightly linked to metabolic state, which in turn is contingent on environmental carbon source.

*AOX1* transcript levels remained constant under all conditions tested. To determine how differential activation of the two *AOX* promoters dictates the abundance of their respective gene products, we compared Aox1 and Aox2 protein levels after growth in AA-containing YPGly medium. Relative Aox2 level was ≥8-fold higher than that of Aox1, when tagged and detected in the same manner (Fig. 2e). Using a strain that co-expresses RFP-tagged Aox2 and a mitochondrial marker protein, Tom70-GFP, we observed that inducible Aox2-RFP localized exclusively within mitochondria (Fig. 2f).

To determine whether an ‘inducible’ *AOX1* could functionally mimic *AOX2*, we used the *AOX2* promoter to drive *AOX1* expression. The resultant hybrid gene was transactivated by AA treatment (Supplementary Fig. 2d), and fully rescued hypersensitivity to AA seen in the *aox*-deletion strain, akin to *AOX2* driven by its native promoter (Fig. 2g).

### Induction of *AOX2* by ETC inhibition requires transcription factors Rtg1 and Rtg3

**Fig. 3:**
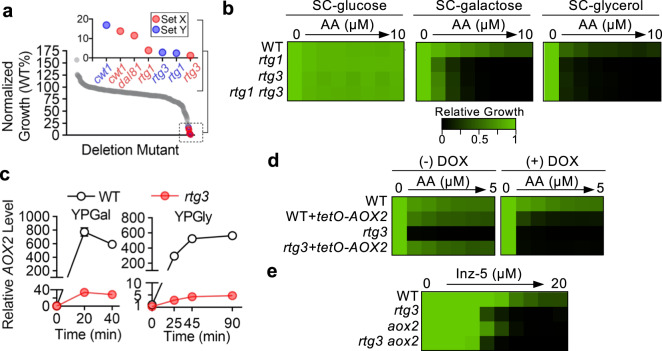
Rtg1 and Rtg3 are required for *AOX2* transactivation induced by AA. **a** Screen of *C. albicans* transcription factor deletion mutants identified *rtg1* and *rtg3* strains as hypersensitive to AA. AA sensitivity (1 μM; SC-galactose) of two independent mutants for each gene (Set X and Set Y) was scored by calculating the ratio of OD_600_ in AA-containing medium to OD_600_ in the vehicle (DMSO)-only medium after incubation for two days. Scores were normalized by setting that of the reference strain to 100%. Mutants strongly inhibited by AA (less than 20% of the wild-type growth) are shown in the inset. The mean of three technical replicates is presented for each data point. **b** *rtg1-* and *rtg3*- deletion mutants are hypersensitive to AA in SC medium with alternative carbon sources. Growth was measured in the specified media supplemented with a 2-fold dilution gradient of AA and is presented in heatmap format with a relative growth scale bar. **c** AA-induced *AOX2* expression requires *RTG3*. Relative *AOX2* transcript levels were measured in strains by RT-qPCR after AA (10 μM) addition to YP with the indicated carbon sources. For normalization, the basal *AOX2* level (time 0) in the WT strain was set to 1. Data represent the mean (SD) of technical triplicates from an experiment representative of biological duplicates with comparable results. **d** Overexpression of *AOX2* in the *rtg3* mutant rescues hypersensitivity to AA. The native *AOX2* promoter in both WT and *rtg3*-deletion strains was replaced by a strong, doxycycline (DOX)-repressible promoter system (*tetO*) to drive *AOX2* over-expression in the absence of DOX. Growth of these strains in SC-galactose medium containing a 2-fold dilution gradient of AA was monitored in the absence or presence of DOX (0.5 μg/mL) and presented in heatmap format. **e** Deletion of *RTG3* and *AOX2*, individually or in combination, have similar effects on sensitivity to Inz-5. WT and mutant strains were tested for growth inhibition by a two-fold dilution gradient of Inz-5 in SC-galactose medium. Results are presented in heatmap format.

In filamentous fungi, transactivation of *AOX* gene expression is mediated by a pair of zinc cluster transcription factors (TFs)^28–30^. In *C. albicans*, however, the TFs controlling *AOX2* induction have not been identified. We screened a *C. albicans* TF-deletion library to identify mutants defective in supporting Aox-dependent growth in SC-galactose medium containing AA. Amongst the 165 TFs represented in the library^31^, *rtg1*- and *rtg3*-deletion strains showed the greatest growth defect (Fig. 3a). Deletion of only two other genes (*CWT1 and DAL81)* reduced growth to <20% of wild-type growth. Except for *dal81*-deletion, no mutants of interest had a growth defect in the absence of AA (Supplementary Fig. 3a).

Rtg1 and Rtg3 belong to the basic helix-loop-helix (bHLH) family of TFs. Further characterization revealed that *rtg1*- and *rtg3-*deletion mutants are hypersensitive to cytochrome pathway inhibition when utilizing diverse non-fermentable carbon sources (Fig. 3b; Supplementary Fig. 3b). Under all conditions tested, homozygous deletion of either *RTG1* or *RTG3* impaired growth to the same extent as homozygous deletion of both genes, consistent with their protein products functioning together as a complex^32,33^. In both YPGal and YPGly, deletion of *RTG3* greatly diminished the activation of *AOX2* expression by AA (Fig. 3c). As a result, Aox2 protein did not accumulate in the *rtg3*-deletion mutant, in marked contrast to the accumulation seen in the wild-type strain (Supplementary Fig. 3c). Unlike Aox2, the level of Aox1 was not controlled by *RTG3* (Supplementary Fig. 3c). Deleting *RTG3* in a *rip1*-deletion background mirrored the effect of deleting *AOX* genes in that it abolished growth on YPGal agar (Supplementary Fig. 3d). Of note, AA treatment modestly increased *AOX2* expression in the *rtg3*-deletion strain (Fig. 3c), suggesting that an Rtg1/Rtg3-independent mechanism may contribute to *AOX2* induction upon inhibition of the cytochrome pathway.

In *C. albicans*, the Rtg1/Rtg3 complex has been implicated in regulating the expression of genes involved in multiple metabolic and signaling pathways^32,33^. To confirm that defective *AOX2* induction, rather than dysregulation of other Rtg1/3 target genes, renders the *rtg3*-deletion strain hypersensitive to AA, we replaced the native promoter of both copies of *AOX2* with a tetracycline-repressible promoter (*tetO*) in both wild-type and *rtg3*-deletion backgrounds. In the absence of doxycycline (a tetracycline analog), *AOX2* expression from the *tetO* promoter was actually greater than AA-induced expression from the native promoter (Supplementary Fig. 3e). The *rtg3* mutant strain overexpressing *AOX2* showed wild-type-like sensitivity to AA in SC-galactose, but hypersensitivity was restored by addition of doxycycline (and therefore repression of *AOX2*, Fig. 3d). Combined deletion of *AOX2* and *RTG3* did not have additive effects on sensitivity to cytochrome pathway inhibition by Inz-5 (Fig. 3e). We conclude that the Rtg1/Rtg3 complex supports AA-resistant alternative carbon source utilization by activating *AOX2* expression.

### Binding of Rtg1/Rtg3 to the *AOX2* promoter is essential for AA-induced transactivation

**Fig. 4:**
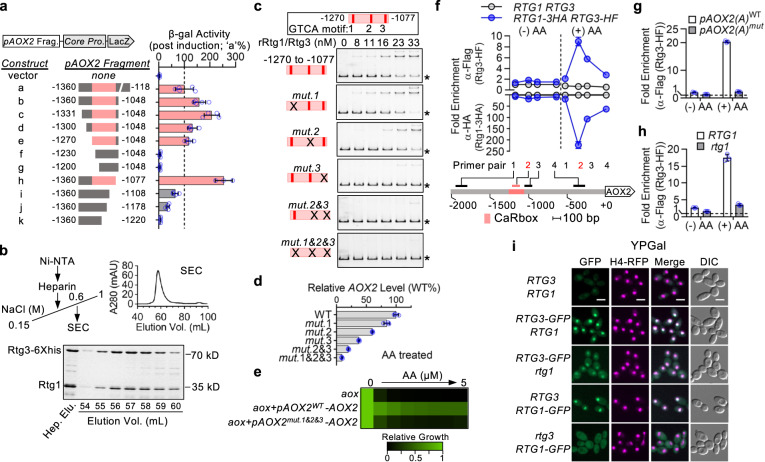
An Rtg1/Rtg3 complex binds the *AOX2* promoter through multiple GTCA motifs to drive induction by AA. **a** Dissection of the *AOX2(A)* promoter (*pAOX2*). Upper, left: Fragments were fused to the *C. albicans CDR2* core promoter (*Core Pro*.) to drive LacZ (β-galactosidase) expression. Lower, left: Promoter fragments (‘a’ to ‘k’) with their nucleotide-positions specified by distance from the start codon. Right: β-gal activity after AA treatment in YPGly. Activity of construct ‘a’ was set to 100%. Red indicates the minimal region required for construct ‘a’-like activity. The mean (SD) obtained from at least three independent strains is presented. **b** Purification of the Rtg1/Rtg3 complex. Top left: Purification scheme. Top right: Elution profile from the size-exclusion chromatography (SEC) column of the applied heparin-binding fraction. Bottom: Coomassie blue staining of SEC fractions from SEC resolved by SDS-PAGE. **c** Identification of critical GTCA motifs within the minimal AA-responsive element defined in **a**. Top: *AOX2* promoter fragment used for gel-shift assays with the three GTCA motifs (1 to 3; 5’ to 3’) highlighted in red. Bottom: Probes with WT sequence or carrying mutation(s) (indicated by ‘x’) in GTCA motifs were incubated with purified Rtg1/Rtg3-6XHis complex at the indicated concentrations. Slower-migrating DNA-protein complex and unbound probe (asterisk) were visualized by SYBR green staining. **d** GTAC motif mutations from **c** were introduced into the full-length promoter at native locus. *AOX2* mRNA levels after AA treatment in YPGal (10 μM; 30 min) were measured and normalized to expression from the WT promoter. Data report mean (SD) of technical triplicates from a representative experiment of biological duplicates. Data for the corresponding biological replicate confirming reproducibility are provided in the source data file. **e** *AOX2* driven by a promoter with disrupted GTCA motifs is unable to rescue hypersensitivity to AA in an *aox*-deletion background in SC-galactose. See relative growth color bar. **f** Binding profile of Rtg1 and Rtg3 at the *AOX2* promoter induced by AA. Top: Strain expressing Rtg1-3XHA and Rtg3-6XHis-3XFlag (‘*RTG1-3HA RTG3-HF*’) and an untagged strain (‘*RTG1 RTG3*’) were grown in YPGal with/without AA (10 μM; 15 min) followed by formalin-fixation for anti-Flag and anti-HA ChIP analyses. Recovery of ChIP signal at tested regions was calculated as the ratio of abundance in IP products to the abundance in IP inputs assessed by qPCR using the numbered primers. Background binding was defined by recovery from the untagged strain without AA. Bottom: Schematic of the tested promoter region. CARbox and CARbox-annealing primer pair 2 are highlighted in red. **g**, **h** Mutation of CARbox GTCA motifs (**g**) or deletion of *rtg1* (**h**) abrogates AA-induced Rtg3 binding to the *AOX2* promoter. AA treatment was performed and background binding (dashed line) was determined as in **f**. **i** Localization of Rtg3-GFP (or Rtg1-GFP) was visualized in YPGal with RFP-tagged Histone H4 (nuclear marker). Strain with untagged Rtg1/Rtg3 was included to assess background GFP signal. Scale bars: 5 μm. **f**–**h** Data represent the mean (SD) of triplicate measurements from one experiment representative of biological duplicates with comparable results.

To determine whether Rtg1/Rtg3 activates the *AOX2* promoter by direct binding, we began by asking which portion(s) of the *AOX2* promoter are required for activation by AA. *AOX2* promoter fragments of variable length were fused to the *CDR2* core promoter sequence followed by a *LacZ* reporter (Fig. 4a). In a reporter strain carrying the full-length *AOX2* promoter construct (‘a’), *LacZ* expression, measured by β-galactosidase activity, was strongly induced by AA treatment in YPGly. Similar or greater induction was observed for all constructs encoding the intact −1270 to −1077 region (p*AOX2* ^−1270 to −1077^, highlighted red in Fig. 4a). Shortening this region from either end decreased inducibility. Indeed, *AOX2* did not rescue AA-hypersensitivity when under control of a mutant promoter that lacks the p*AOX2* ^−1270 to −1077^ fragment (Supplementary Fig. 4a). To test whether the Rtg1/Rtg3 complex can bind to this fragment in vitro, we purified recombinant complex from *Escherichia coli* cells that co-express Rtg1 and tagged Rtg3. The two proteins co-purified through all chromatography steps and co-eluted at a stoichiometry of ~1:1, suggesting equimolar presence of the two components in a stable complex (Fig. 4b). This complex bound double-stranded DNA encoding the p*AOX2* ^−1270 to −1077^ sequence, decreasing its mobility in gel-shift assays (Fig. 4c).

A genome-wide chromatin immunoprecipitation (ChIP) study previously reported that *C. albicans* Rtg1 and Rtg3 share the same consensus binding sequence^34^. The motif at the 5′ end of this consensus sequence is highly similar to the ‘GTCAC’ motif (R box) reported to be bound by *Saccharomyces cerevisiae* Rtg1/Rtg3^35^. Within the *C. albicans* p*AOX2* ^−1270 to −1077^ fragment, we identified three GTCA motifs, which differentially contributed to Rtg1/Rtg3 binding in vitro. Interaction was strongly destabilized by mutating the GTCA motif closest to the 3′ end of the p*AOX2* ^−1270 to −1077^ probe (*mut.3*; Fig. 4c). Disruption of the middle GTCA motif (*mut. 2*) also decreased binding, but to a lesser extent. A greater decrease in affinity was observed when both sites were mutated (*mut. 2&3*). Mutating the motif closest to the 5′ end of the probe (*mut.1*) did not decrease Rtg1/Rtg3 binding. When these GTCA mutations were introduced into the native *AOX2* promoter in cells, the inducibility of each mutant correlated well with its relative in vitro affinity for Rtg1/Rtg3 in gel-shift assays (Fig. 4c, d). *AOX2* induction driven by a promoter with all three GTCA motifs disrupted was only 10–15% of the induction seen with a wild-type promoter (Fig. 4d) and this level of induction was insufficient to support growth in SC-galactose medium containing AA (Fig. 4e).

We also directly compared the effects on *AOX2* induction by AA of deleting *RTG3* versus mutating the GTCA motifs. Loss of *RTG3* resulted in a greater decrease in induction, suggesting that the *AOX2* promoter may have additional Rtg1/Rtg3 binding site(s), which play a minor role in mediating transactivation (Supplementary Fig. 4b). Interestingly, mutating the GTCA motifs further decreased *AOX2* induction in the *rtg3*-deletion mutant (Supplementary Fig. 4b), suggesting these motifs may also be involved in transactivation through an Rtg1/Rtg3-independent mechanism. Going forward, we refer to the −1270 to −1077 region of the *AOX2* promoter as CARbox (*C. albicans* Antimycin Responsive box).

### Inhibiting respiration induces Rtg1/Rtg3 binding to the CARbox

To learn whether increased promoter occupancy by Rtg1/Rtg3 is involved in *AOX2* transactivation, we performed chromatin immunoprecipitation (ChIP) assays (Fig. 4f). The epitope tags we introduced to enable this approach did not interfere with Rtg1/Rtg3 function in activating *AOX2* expression (Supplementary Fig. 4c). In cells grown in YPGal, ChIP signals were close to background levels, which were determined in a strain expressing untagged proteins (Fig. 4f). Upon exposure to AA, however, ChIP signals for both Rtg1-3XHA and Rtg3-HF increased across the *AOX2* promoter concordantly. The greatest increase was at the CARbox region for both proteins (Fig. 4f). Based on in vitro and functional analyses, we predicted that Rtg1 and Rtg3 would bind as a complex to GTCA motifs within the CARbox. Indeed, mutating GTCA motifs or deleting *rtg1* abolished AA-induced binding of Rtg3-HF to the CARbox (Fig. 4g, h). Substituting glucose for galactose reduced Rtg1 binding at the CARbox upon AA treatment, indicating glucose represses *AOX2* induction upstream of Rtg1/Rtg3 binding to its promoter (Supplementary Fig. 4d).

In the model yeast *S. cerevisiae*, Rtg1/Rtg3 orthologues localize to the cytosol under basal conditions and must translocate into the nucleus to activate target gene expression^36^. In the fungal pathogen *C. albicans*, however, we found different biology. Using a strain that co-expresses from their native promoters GFP-tagged Rtg3 and RFP-tagged Histone H4 (nuclear protein encoded by *HHF1*), we found that Rtg3-GFP co-localized with H4-RFP in the nucleus, even under non-inducing conditions (Fig. 4i). We confirmed that GFP-tagging did not interfere with Rtg3 function in regulating *AOX2* expression (Supplementary Fig. 4c). We repeated these experiments using GFP-tagged Rtg1 and obtained the same results that growth in other carbon sources or AA treatment did not alter Rtg1/Rtg3 localization (Supplementary Fig. 4e, f). Additional experiments revealed that nuclear localization requires Rtg1 and Rtg3 to be in complex. Under all conditions tested, Rtg1-GFP was excluded from the nucleus in the absence of Rtg3, whereas deletion of *RTG1* decreased but did not eliminate nuclear localization of Rtg3-GFP (Fig. 4i; Supplementary Fig. 4f).

### Maximal transactivation requires a second TF complex consisting of Cwt1 and Zcf11

**Fig. 5:**
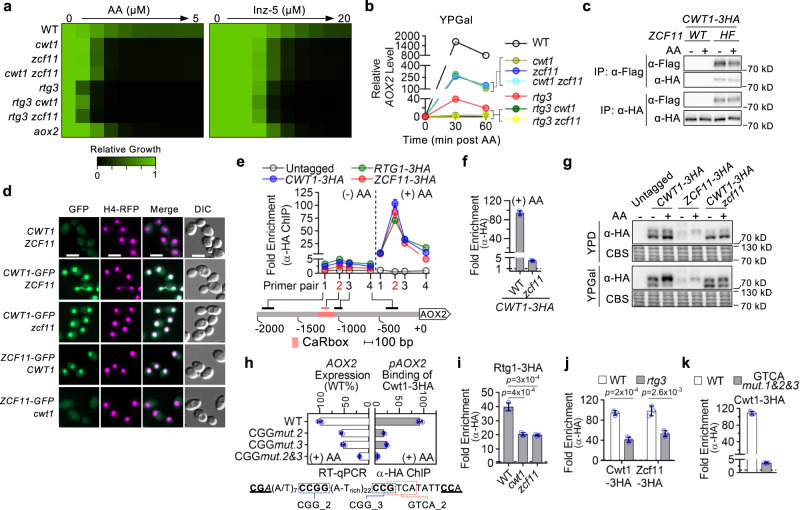
Cwt1 and Zcf11 comprise a transcription factor complex required for AA-induced *AOX2* transactivation. **a** Strains were tested for sensitivity to AA or Inz-5 in SC-galactose. **b** Relative *AOX2* transcript levels were measured following AA addition (10 μM) in YPGal. Basal *AOX2* expression (time 0) in the WT background was set to 1. **c** *C. albicans* expressing Cwt1-3XHA with native Zcf11 or Zcf11-6XHis-3XFlag (Zcf11-HF) were grown in YPGal with/without AA treatment. Anti-HA and anti-Flag immunoprecipitation (IP) products were analyzed by immunoblotting. **d** Localization of Cwt1-GFP and Zcf11-GFP were monitored by fluorescence microscopy. Scale bar: 5 μm. **e** Binding profile of Cwt1-3XHA, Zcf11-3XHA, or Rtg1-3XHA at the *AOX2* promoter before and after AA treatment (10 μM; 15 min; YPGal) were analyzed by anti-HA ChIP using the primer set in Fig. 4f. Background signal determined using an untagged WT strain without AA treatment. **f** Cwt1-3XHA binding at the CARbox was compared between WT and *zcf11*-deletion strains. AA treatment and background signal assessment were performed as in **e**. **g** WT or *zcf11*-deletion strains expressing Cwt1-3XHA or Zcf11-3XHA were treated with AA (10 μM; 15 min) in YPD or YPGal. Blots were probed using anti-HA antibody. CBS: Coomassie Blue staining of the blot showing comparable loading. **h** *AOX2* promoters with mutation(s) in the CGG motif 2 or (and) motif 3 (see Supplementary Fig. 5f) in a strain expressing Cwt1-3XHA. Top left: AA-induced *AOX2* expression from the WT and mutant promoters. Top right: Cwt1-3XHA binding at CARbox upon AA treatment in YPGal (10 μM; 15 min) by α-HA ChIP. Bottom: DNA sequence flanking CGG motifs 2 and 3. **i**, **j** AA-induced CARbox binding of Rtg1/Rtg3 (or Cwt1/Zcf11) as measured by ChIP was compared between WT and mutant strains after AA treatment (10 μM; 15 min; YPGal). *p* values: two-tailed *t* test (*n* = 3). **k** Mutation of the GTCA motifs abolish AA-induced Cwt1 binding at CARbox, as assessed by ChIP. **b**, **e**, **h** (left) present mean (SD) of technical triplicates from an experiment representative of biological duplicates. Data for the corresponding biological replicates are in source data file. **f**, **h** (right), **i**, **j**, **k** present mean (SD) of biological triplicates.

In our TF-mutant screen, two independent mutants with deletion of *CWT1* were also identified as hypersensitive to AA, though to a lesser extent than *rtg1*- or *rtg3*-deletion mutants (Fig. 3a). In multiple filamentous fungi, a Cwt1 homolog acts in complex with another zinc cluster TF to regulate alternative oxidase expression^28–30^. In *C. albicans*, *ZCF11* encodes a predicted Cwt1 binding partner. A *zcf11* mutant was not included in the TF-deletion collection that we screened. When utilizing galactose or glycerol as carbon source, we observed that a *cwt1-*deletion strain and a *zcf11*-deletion strain that we constructed showed similar degrees of hypersensitivity to Complex III inhibitors AA or Inz-5 (Fig. 5a; Supplementary Fig. 5a), which correlated with ~80% lower *AOX2* transcript level after AA treatment in YPGal or in YPGly (Fig. 5b; Supplementary Fig. 5b). Double deletion of *CWT1/ZCF11* phenocopied individual homozygous deletion mutants, suggesting that *C. albicans* Cwt1 and Zcf11 likely function as a heterocomplex to regulate Aox transactivation. Strains with either *CWT1* or *ZCF11* deletion were also hypersensitive to AA on agar medium containing non-fermentable carbon sources (Supplementary Fig. 5a). Of note, the *cwt1*-deletion mutant was defective in utilizing these carbon sources even in the absence of Complex III inhibition. The *zcf11*-deletion strain, however, did not show a similar phenotype, suggesting that Cwt1 has Zcf11-independent function(s) in supporting alternative carbon source utilization. Deletion of *CWT1* or *ZCF11* in the *rtg3*-deletion background virtually eliminated *AOX2* induction by AA (Fig. 5b), but its effect on sensitivity to AA was masked by the overwhelming hypersensitivity caused by *rtg3*-deletion alone (Fig. 5a).

To further investigate how Cwt1/Zcf11 responds to AA in transactivating *AOX2*, we constructed strains expressing various tagged versions of these proteins from both copies of their native promoters. Modified strains showed wild-type-like growth sensitivity to AA and expressed *AOX2* at levels comparable to those of untagged strains under both non-inducing and AA-inducing conditions (Supplementary Fig. 5c, d). By co-immunoprecipitation, Cwt1 was found to interact with Zcf11 not only after AA treatment, but also under non-inducing conditions (Fig. 5c). Microscopy of GFP-tagged strains revealed constitutive nuclear localization of Cwt1-GFP and Zcf11-GFP under non-inducing growth conditions (Fig. 5d). Therefore, respiration inhibition does not activate the Cwt1/Zcf11 complex by inducing heterodimerization or nuclear translocation.

We next used ChIP assays to determine whether AA treatment induces Cwt1/Zcf11 binding to the target gene-promoter. Under non-inducing conditions, Cwt1 and Zcf11 occupancy at the *AOX2* promoter was similar to background but greatly increased following AA treatment (Fig. 5e). With peak occupancy at the CARbox, the ChIP binding profile of Cwt1/Zcf11 across the *AOX2* promoter was similar to that of Rtg1. Deletion of *ZCF11* abolished Cwt1 binding at the CARbox without changing the protein level or nuclear localization of Cwt1 (Fig. 5d, f; Supplementary Fig. 5e). Both microscopy and immunoblotting indicated that tagged Zcf11 proteins were less abundant than Cwt1 when fused to the same tags, and they became barely detectable in a *cwt1*-deletion background (Fig. 5d; Supplementary Fig. 5e). Collectively, these results suggest co-dependence for Cwt1 and Zcf11 binding to the CARbox to activate *AOX2* transactivation. By immunoblotting, Cwt1-3XHA migrated as two major bands of the expected molecular size. The relative abundance of the two forms changed with carbon source and AA treatment shifted almost all of the Cwt1-3XHA signal to the slower-migrating form (Fig. 5g). Glucose as the carbon source or deletion of *zcf11* repressed these shifts in mobility, suggesting a correlation between gel mobility and activation state for Cwt1. AA treatment also induced a mobility shift for Zcf11-3XHA, which was more subtle and independent of the carbon source (Fig. 5g). Defining the molecular basis for these changes in mobility will require additional investigation.

### Cwt1/Zcf1 and Rtg1/Rtg3 bind the CARbox in a cooperative manner

Homo- or heterodimerized zinc cluster TFs, such as Cwt1/Zcf11, usually recognize a pair of CGG triplets in the same or inverted orientation spaced by nucleotides of variable length and sequence^37^. In filamentous fungi, Cwt1/Zcf11 homologs interact with the *AOX* promoter through a CGG(N)_7_CGG motif^28,29,38^, which is absent from the CARbox or other regions of the *C. albicans AOX2* promoter. Instead, the CARbox encodes three CGG triplets separated by 31 or 22 bp. Individual mutation of either of the two CGG motifs closest to the 3′ end of CARbox decreased AA-induced *AOX2* expression by ~50% (Supplementary Fig. 5f). When both sites were mutated, AA-induced *AOX2* expression dropped to ~15% of that driven by a wild-type promoter (Fig. 5h). By ChIP, we confirmed that *AOX2* transactivation defects caused by CGG mutations correlated with reduced Cwt1 binding at the CARbox (Fig. 5h). Therefore, CGG motifs 2 and 3 are likely the binding sites for AA-activated Cwt1/Zcf11. Interestingly, each of the two CGG motifs forms a CGG(N)7CGG-like motif with the flanking sequence (underlined in Fig. 5h).

With both heterocomplexes required for maximal *AOX2* transactivation, we wondered how Rtg1/Rtg3 and Cwt1/Zcf11 might interact with each other. Comparing the expression and localization of each complex before and after the deletion of the other, we found no interdependency between the two complexes for maintenance of the other’s protein level or nuclear localization (Supplementary Fig. 5g–j). Immunoprecipitation did not support inter-complex interaction to form a super-complex under either non-inducing or inducing conditions (Supplementary Fig. 5k). By ChIP, however, ~50% of Rtg1 binding was sensitive to deletion of *CWT1* or *ZCF11*, while the other half was resistant (Fig. 5i). Similarly, binding of Cwt1/Zcf11 at the CARbox showed approximately equal contributions from Rtg3-dependent and Rtg3-independent components (Fig. 5j). Such interdependence supports a model in which the two complexes are independently activated, but bind the *AOX2* promoter in a cooperative manner to achieve maximal transactivation. Consistent with this model, Cwt1-3HA in an AA-treated *rtg3*-deletion strain still shifted in mobility as a potential marker of activation (Supplementary Fig. 5j). Lastly, we asked how mutation of the GTCA motifs, which support Rtg1/Rtg3 binding, affect Cwt1/Zcf11 binding at the CARbox. Mutation of GTCA motif 2 partially disrupts the overlapping CGG motif 3 (Fig. 5h). Therefore, we predicted that as a consequence of losing Rtg1/Rtg3 binding and compromising a direct binding site for Cwt1/Zcf11, mutation of all three GTCA motifs would abolish Cwt1/Zcf11 binding. This prediction was confirmed by ChIP (Fig. 5k) and explains the observation that GTCA motif mutations decrease the *RTG3*-independent transactivation of *AOX2* induced by AA (Supplementary Fig. 4b).

### Cyanide treatment activates *AOX2* expression through multiple mechanisms

**Fig. 6:**
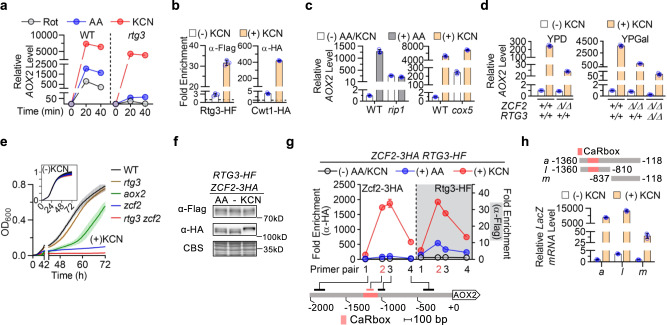
Zcf2 is the primary activator of *AOX2* in response to cyanide. **a** Cyanide induces *AOX2* primarily through an *RTG3*-independent mechanism. Levels of *AOX2* in WT and *rtg3*-deletion strains growing in YPGal were assessed following treatment with rotenone (Rot; 2.5 μM), AA (10 μM), or potassium cyanide (KCN; 1 mM). **b** Strains expressing Rtg3-6XHis-3XFlag or Cwt1-3XHA were treated with KCN (1 mM; 15 min) in YPGal. Relative CARbox binding was assessed by anti-Flag or anti-HA ChIP. **c** KCN induces *AOX2* in a *cox5*-deletion strain. *rip1*-deletion or *cox5*-deletion strains were treated with AA (10 μM; 30 min) or KCN (1 mM; 20 min). Relative *AOX2* expression was measured and compared to the WT strain. **d** *AOX2* expression was measured in WT (+/+) versus *zcf2*-deletion (Δ/Δ) backgrounds after KCN treatment (1 mM; 20 min) in YPD (left) or YPGal (right). A *zcf2*/*rtg3*-deletion strain was evaluated to examine the role of *RTG3* in regulating Zcf2-independent induction in YPGal. **e** Growth of the indicated strains in YPGly (inset) with or without 2 mM KCN were assessed by serial measurements of OD_600_. Data report mean (SD) from technical quadruplicates from a representative experiment of biological duplicates. **f** A strain expressing Rtg3-HF and Zcf2-3XHA was treated with AA (10 μM) or KCN (1 mM) for 15 min in YPGal. Lysates were resolved by SDS-PAGE and blots probed with anti-Flag and anti-HA antibodies. **g** Zcf2 binds the *AOX2* promoter upon KCN exposure. Binding profile of Zcf2-3XHA and Rtg3-HF at the *AOX2* promoter were assessed by ChIP after 15-min treatment with AA (10 μM) or KCN (1 mM) in YPGal. Relative binding was determined using the same primers depicted in Fig. 4f. **h** Indicated promoter fragments were introduced into the reporter system described in Fig. 4a and tested for response to cyanide in a *rtg3*-deletion background. *LacZ* transcript levels were assessed before and after cyanide treatment (1 mM; 15 min; YPGal). At least two independent strains were tested for each construct and yielded consistent results. **a**–**d**, **g**, **h** present mean (SD) of technical triplicates from an experiment representative of biological duplicates with comparable results. See source data file.

We next examined whether other conditions that block classical respiration induce *AOX2* through the same transcriptional mechanism. Similar to AA, myxothiazol, another Complex III inhibitor, requires Rtg1/3 function to potently induce *AOX2* expression (Supplementary Fig. 6a). Decreasing oxygen availability by shifting cells grown in respiratory medium to an anaerobic chamber also resulted in *AOX2* transactivation in a largely Rtg1/Rtg3-dependent manner (Supplementary Fig. 6b). Cyanide inhibits Complex IV and is a classical activator of *AOX2* expression^15^. Compared with AA or the complex I inhibitor rotenone, KCN triggered the most robust *AOX2* transactivation (Fig. 6a). Surprisingly, unlike rotenone or AA, KCN activated *AOX2* expression primarily through an *RTG3*-independent mechanism. Deletion of *CWT1* in an *rtg3*-deletion background further decreased induction by AA as expected but did not alter induction by KCN (Supplementary Fig. 6c). To confirm activation of the Rtg1/Rtg3-Cwt1/Zcf11 pathway by KCN, we assessed Rtg1 and Cwt1 binding at the *AOX2* promoter by ChIP. Both transcription factors showed strong binding at the CARbox after KCN treatment (Fig. 6b), which was accompanied by a mobility shift of Cwt1 as detected by immunoblotting (Supplementary Fig. 6d). Therefore, cyanide appears to increase *AOX2* expression through an additional, respiration-independent mechanism(s). Supporting this possibility, deletion of the Complex IV subunit gene *COX5* did not prevent *AOX2* induction by KCN (Fig. 6c). In contrast, deletion of the Complex III subunit gene *RIP1* rendered *AOX2* expression unresponsive to AA.

### Zcf2 acts as a cyanide-responsive regulator of *AOX2* expression

Seeking an as-yet unidentified KCN-responsive regulator of *AOX2*, we prioritized three candidates from the zinc cluster TF family: Cta4, Stb5, and Zcf2. The first two proteins are known to increase *AOX2* expression when fused with a potent transcriptional activation domain^39^, and Zcf2 was chosen for its proposed role in supporting optimal *AOX2* induction by reactive-sulfur species (RSS)^40^. Measuring *AOX2* induction by KCN in deletion mutants of each gene, we found that induction in YPD was not affected by deletion of *CTA4* or *STB5* (Supplementary Fig. 6e), but was greatly decreased in the *zcf2*-deletion strain (Fig. 6d). In YPGal, Zcf2 was responsible for ~85% of KCN-induced *AOX2* expression (Fig. 6d), but dispensable for *AOX2* induction by AA (Supplementary Fig. 6f). Therefore, Zcf2 is likely the previously unknown *AOX2* transactivator that senses KCN, independent of its inhibition of Complex IV, and although less important, the Rtg1/Rtg3 pathway contributes to maximal *AOX2* induction by KCN in cells utilizing galactose (Fig. 6a). Deleting *RTG3* in the *zcf2*-deletion background further compromised *AOX2* induction by KCN in YPGal (Fig. 6d). Given a report that *AOX2* is required for KCN-resistant growth in medium containing alternative carbon sources^15^, we tested whether deleting *ZCF2* and/or *RTG3* to impair *AOX2* transactivation would sensitize cells to growth inhibition by cyanide. Deletion of *AOX2* delayed exponential growth in KCN-containing YPGly medium (Fig. 6e). The *rtg3-*deletion strain showed wild-type-like growth in the same medium, consistent with Rtg3’s minor contribution to *AOX2* induction by KCN. Much greater KCN sensitivity was observed when *ZCF2* was deleted in either a wild-type or *rtg3*-deletion background, suggesting that in addition to *AOX2*, other KCN-responsive genes are regulated by Zcf2 (Fig. 6e).

We next constructed a strain expressing tagged Zcf2 from both copies of the native promoter. It showed a ~two-fold increase in *AOX2* expression under both non-inducing and cyanide-inducing conditions, suggesting that C-terminal tagging may slightly potentiate Zcf2 transcriptional activating activity, but does not impair its general function (Supplementary Fig. 6g). Tagged Zcf2 was detected at the expected molecular weight with a mobility shift seen after KCN but not AA treatment, suggesting a relationship between gel mobility and activation state (Fig. 6f). Using a strain that expresses differentially tagged Zcf2 and Rtg3, we found that under non-inducing conditions, binding of Zcf2 and Rtg3 at the *AOX2* promoter were close to background level as monitored by ChIP (Fig. 6g). Both KCN and AA treatment increased Rtg3-HF ChIP signal as expected, while Zcf2 binding to the *AOX2* promoter was selectively induced by cyanide. Deletion of *RTG3* did not change either the level or profile of Zcf2 binding, consistent with the conclusion that Rtg3 plays a minor role in *AOX2* induction by KCN (Supplementary Fig. 6h). The binding profile of Zcf2 was similar to that of Rtg3, except that the Zcf2-binding peak appeared to be broader, extending from the CARbox into the 3’ flanking region (Fig. 6g). When tested in our *LacZ* reporter system (Fig. 4a), the ~500 bp region that showed significant Zcf2 binding (region −1360 to −810, which is approximately covered by primer pairs 1b to 3b in Supplementary Fig. 6h) was necessary and sufficient to drive reporter transactivation in response to cyanide (Fig. 6h).

Attempts to identify the Zcf2 binding element(s) more precisely were not successful. Prior to this study, a bioinformatic analysis of the Zcf2 regulon under RSS conditions was also unable to achieve a definitive binding consensus^40^. The only experimentally-confirmed element essential for Zcf2-mediated transactivation is a CCTCGG motif at a known RSS-responsive promoter^40^. The −1360 to −810 region contains such a motif. However, mutating it alone or in combination with all ten of the other CGG triplets within the −1360 to −810 region did not disrupt *AOX2* induction by cyanide (Supplementary Fig. 6i). Immunoprecipitation did not detect a stable interaction between 3XHA-tagged Zcf2 and 6XHis-3XFlag-tagged Zcf2 or Cwt1 under either normal or KCN-inducing conditions, suggesting that Zcf2 likely functions as a monomer (Supplementary Fig. 6j).

### CARbox-region elements mediate *AOX2* induction by diverse stressors to enable virulence

**Fig. 7:**
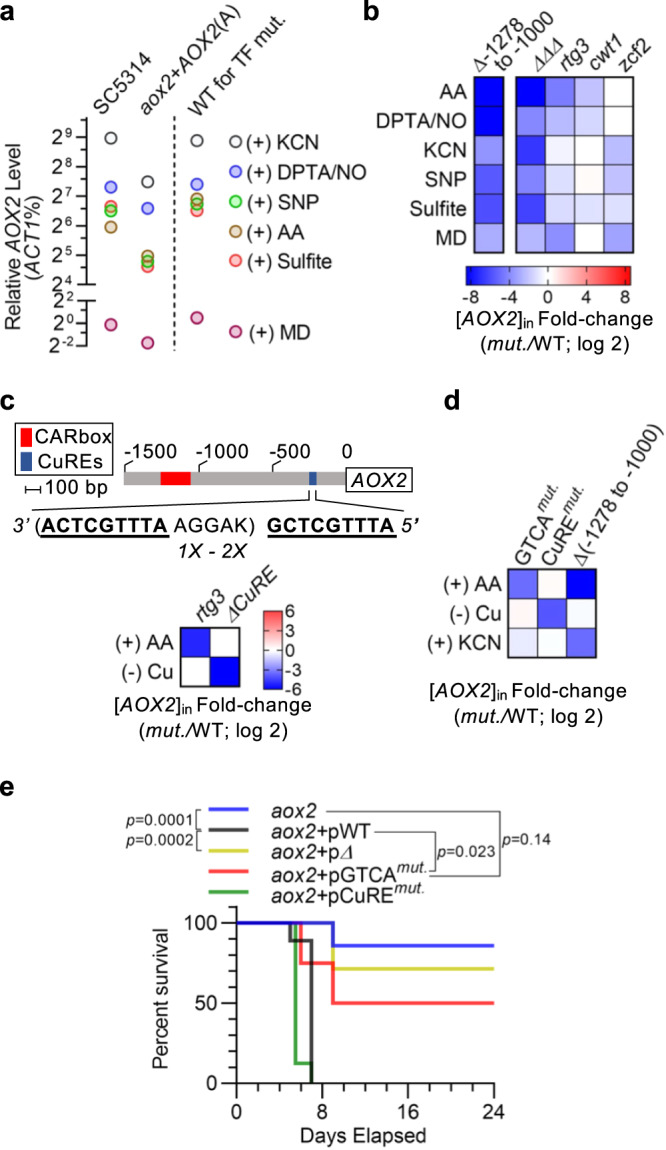
Defined elements within the *AOX2* promoter are required for response to specific stressors and support *C. albicans* virulence. **a** Strains were treated with: AA (10 μM), KCN (1 mM), menadione (MD, 0.8 mM) in YPGal, sodium sulfite (0.1 mM for SC5314 and *aox2* + *AOX2(A)* or 0.2 mM for transcription factor (TF) mutant reference strain) in YPGal (pH 4.0), sodium nitroprusside (SNP, 1 mM), or DPTA NONOate (DPTA/NO, 1 mM) in YPGal (pH 7.2). Exposure time was 60 min for MD and 20 min for all other agents. Relative *AOX2* level after induction is presented as percentage of *ACT1* level (*ACT1*%) from the same sample and plotted in log_2_ scale. *AOX2* expression varied little with pH (*ACT1*% range: 2^−6.5^ to 2^−8.0^). **b** Left: Comparison of *AOX2* levels post induction ([*AOX2*]_in_) between strains with an intact or mutant (nucleotides −1278 to −1000 deleted) promoter. Right: Comparison of *AOX2* induction between strains with individual or triple TF-deletion versus the WT strain. Treatments were performed as in **a**. [*AOX2*]_in_ ratios (log_2_) are presented in heatmap (see scale bar). Effects on [*AOX2*]_in_ are presented in the same manner throughout this figure. **c** Top: Schematic of *AOX2* promoter depicting Mac1-binding consensus sequence (bold font). *AOX2(B)* encodes two copies of ‘KAGGAATTTGCTCA’ (K: G or T), while the A allele encodes one. Bottom: Mutants with *rtg3*-deletion or altered CuREs within the *AOX2* promoter were cultured in the presence of AA (10 μM; 20 min) or in the absence of copper (-Cu). [*AOX2*]_in_ was compared between the mutant and WT strain. **d** *AOX2(A)* promoters with mutation of GTAC motifs, CuREs (ATTTGCTCA to ATTTtaTaA), or with deletion of nucleotides −1278 to −1000 were used to drive *AOX2(A)* in *aox2*-deletion strain. Relative *AOX2* induction by AA (10 μM; 20 min), KCN (1 mM; 20 min), or copper starvation in mutant strains were compared to WT. See scale bar in **b**. **e** Mutation of *AOX2* promoter impairs *C. albicans* virulence. Survival of mice (*n* = 8) after challenge by each strain was compared to parental *aox2*-deletion strain. *p* value: Mantel-Cox test (*n* = 8). **b**–**d** present mean of technical triplicates from an experiment representative of biological duplicates. See source data file.

In animal hosts, *C. albicans* encounters complex nitrosative and oxidative stresses^41^. These types of insults, when mimicked in vitro, activate *AOX2* expression^15,42,43^. We found that treatment with the nitric oxide donors sodium nitroprusside (SNP) or DPTA NONOate (DTPA/NO) induced upregulation of *AOX2* to a similar extent as AA, but less than KCN (Fig. 7a). Sulfite as a source of RSS also induced *AOX2* expression to a level comparable to AA. Superoxide anion donor menadione only increased *AOX2* expression to a level ~2% of that observed with AA. Deletion of *AOX2* did not confer hypersensitivity to menadione or hydrogen peroxide (Supplementary Fig. 7). In an *aox2*-deletion strain that carries a re-introduced wild-type *AOX2(A)* allele, native-like inducibility by each stress was observed, although relative magnitudes were lower, likely because the strain only carries a single allele of *AOX2* (Fig. 7a). Having characterized responses mediated by the wild-type *AOX2* promoter to host-relevant chemical stresses, we compared these responses to those driven by a mutant promoter in which *pAOX2* ^*−1278 to −1000*^ had been deleted. This promoter mutation crippled induction in response to all stressors (Fig. 7b). Therefore, *pAOX2* ^*−1278 to −1000*^ is required not only for induction by AA/KCN, but also for induction by other challenges. Indeed, combined homozygous deletion of all three TF genes found to interact with the promoter in this region (*RTG3*, *CWT1*, and *ZCF2*) also impaired transactivation by all six stressors (Fig. 7b).

Examining single deletion mutants, we found that, akin to induction by AA/KCN, maximal response to SNP, DPTA/NO, or menadione was also mediated by multiple TFs (Fig. 7b). Interestingly, DPTA/NO and SNP showed differential TF requirements for activating *AOX2* expression. Induction by DPTA/NO depended on Rtg1/Rtg3 and Cwt1/Zcf11, while induction by SNP was more sensitive to the deletion of *ZCF2*, suggesting that SNP transactivates *AOX2* through both NO- and cyanide-mediated stress. Both the Rtg1/Rtg3 and Zcf2 were essential for induction by menadione, but Cwt1/Zcf11 appeared to be dispensable. Regarding reactive-sulfur stress, single deletion of each transcription factor gene modestly decreased *AOX2* induction by sulfite, but deletion of all three genes or the *pAOX2* ^*−1278 to −1000*^ region largely abolished *AOX2* induction by sulfite. All considered, we conclude that a regulatory hub consisting of the *pAOX2* ^*−1278 to −1000*^ promoter sequence and the TFs Rtg1/Rtg3, Cwt1/Zcf11, and Zcf2 integrate signals from diverse environmental stresses to activate *AOX2* expression.

In addition to the *AOX2* transactivators identified here, the copper-sensing transcription factor Mac1 has previously been reported to drive *AOX2* induction upon copper starvation^44^. The *AOX2* promoter contains multiple copies of conserved Mac1-binding elements (copper-responsive elements; CuREs)^45,46^, which are located ~1 kb downstream of the CARbox. We found that deletion of these CuREs abolished *AOX2* induction by copper starvation without affecting induction by AA, indicating that Mac1 mediates a distinct *AOX2-*inducing response (Fig. 7c).

**Fig. 8:**
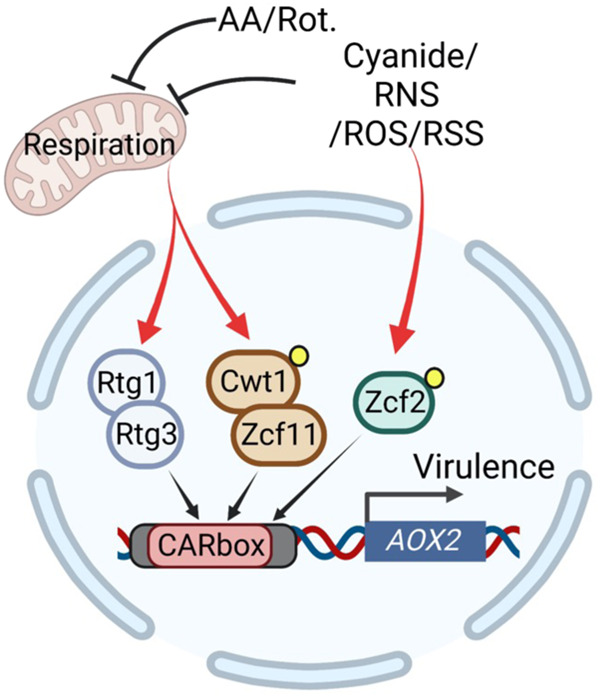
Model for transcriptional control of *AOX2* in *C. albicans*. Under growth conditions requiring respiration, inhibition of the classical ETC triggers cooperative binding of the Rtg1/Rtg3 and the Cwt1/Zcf11 transactivator complexes to the CARbox. Highly specific ETC inhibitors (e.g., AA and rotenone) activate *AOX2* primarily through this pathway. More promiscuous inhibitors such as cyanide, nitric oxide, and reactive oxygen or sulfur species can activate *AOX2* expression through a second Zcf2-meditated pathway. This pathway requires binding of Zcf2 to the *AOX2* promoter but is less dependent on cellular metabolic state. Yellow dots indicate potential changes in the post-translational modification of Cwt1 and Zcf2 under activating conditions. This image was created with BioRender.com.

Finally, to determine how disrupting each of the stress-selective *AOX2* transactivation mechanisms identified in vitro would alter the virulence of *C. albicans* upon systemic infection of mice, we assembled a panel of strains created in an *aox2* homozygous deletion mutant that was complemented with *AOX2* under control of (1) wild-type promoter, (2) promoter with mutated GTCA motifs to selectively compromise induction through Rtg1/Rtg3 and Cwt1/Zcf11, (3) promoter with deletion of nucleotides −1278 to −1000 (*pAOX2* ^−*1278 to −1000*^) to disrupt Rtg1/Rtg3- Cwt1/Zcf11- and Zcf2-mediated activation, and (4) promoter with mutated CuREs to eliminate Mac1-mediated activation. Each mutant strain showed the expected condition-selective decrease in *AOX2* induction compared to the wild-type-complemented strain when treated with AA, KCN, or upon growth in low-copper medium (Fig. 7d). Following intravenous infection of mice, we found that virulence of the *aox2*-deletion strain was restored to near-parental level by re-introduction of a single copy of *AOX2* driven by its wild-type promoter or a mutant promoter with disrupted CuREs (Fig. 7e). In contrast, complementation with mutated GTCA motifs or deleted *pAOX2* ^−*1278 to −1000*^ did not restore virulence, with a stronger defect observed for the *pAOX2* ^−*1278 to −1000*^ deletion mutant. Overall, findings support a model in which both Rtg1/Rtg3- Cwt1/Zcf11- and Zcf2-dependent pathways contribute to *C. albicans* virulence by enabling *AOX2* induction in response to the environmental challenges, which must be overcome to cause lethal infection (Fig. 8).

## Discussion

Metabolic flexibility enables microbial pathogens to invade hostile host environments and cause disease. Here, we find that the inducible expression of the alternative oxidase, *AOX2* contributes greatly to the flexibility and virulence of *C. albicans*. Our study indicates that the virulence defect caused by loss of *AOX2* expression is associated with increased leukocyte accumulation at sites of infection, either due to increased recruitment or greater survival of the responding immune cells. The difference in host response early in the course of infection when fungal burdens are comparable presumably leads to enhanced clearance of the mutant as time progresses and greater overall survival. Although the precise mechanism(s) remains to be defined, increased recognition of *C. albicans* by the host would be consistent with previous reports that Aox activity plays an important role in the maintenance of cell wall architecture^16^ and glucan masking under hypoxic conditions^17^.

Aox activity is found in a wide variety of organisms ranging from plants to protozoans to fungi, but not in humans^47^. Although relatively inefficient in generating energy, alternative respiration allows for the direct transfer of electrons from ubiquinol to oxygen. Thus, fungi with *AOX* genes can bypass blockades in the classical cytochrome pathway to maintain redox balance and TCA cycle function and in turn, utilize alternative carbon sources. Besides *C. albicans*, the other major human fungal pathogens also have an *AOX* encoded in their genomes. Consistent with our findings, the virulence of *C. neoformans* is impaired by deletion of its *AOX*^48^. However, previous studies with *Aspergillus fumigatus* found no virulence defect associated with *AOX* deletion^49^. This disparity could be due to differences in the mouse models used to assess virulence. The *A. fumigatus* studies were performed in immunocompromised mice, while our studies and those with *C. neoformans* were performed in immunocompetent mice. At least in *C. albicans*, we find that fungal Aox activity reduces the accumulation of innate immune cells in organs of immunocompetent animals, which are known to generate both reactive oxygen and nitrogen species that damage classical ETC pathway components. After phagocytosis by murine macrophages, *AOX2* transcript level rapidly increases even before upregulation of genes associated with filamentation, a process coupled to macrophage killing and escape^23^, highlighting that induction of *AOX2* is part of the primary transcriptional response of *C. albicans* to engulfment by host phagocytes. Alternatively, the very different pathogenetic mechanisms used by these organisms may impose differential requirements for Aox activity in enabling them to cause disease. The surprisingly complex network of transcription factors that we uncovered in *C. albicans* provides clues as to the specific challenges encountered during invasive infection that Aox2 may help this fungus overcome. With the relevant transcriptional activators now identified, techniques such as Calling card-Seq could be used in future work to learn where and when *AOX2* expression is induced during the progression of invasive infection and in other commensal or disease models^50^.

In several constitutively filamentous fungi, functional homologs of the zinc cluster transcription factor complex Cwt1/Zcf11 play an important role in the upregulation of *AOX* expression, as we now show for *C. albicans*^29,38,51^. Previously unknown, however, is the role of the Rtg1/Rtg3 complex that we define here. The retrograde response pathway involving this TF complex in *S. cerevisiae* allows signal(s) generated by mitochondria to drive altered nuclear gene expression, but it has not been previously implicated in the control of *AOX* expression^52^. Within the *AOX2* promoter of *C. albicans*, binding sites for the Cwt1/Zcf11 and Rtg1/Rtg3 complexes are in close proximity and located ~1.2 kb upstream of the transcriptional start site. Interestingly, the 1.2 kb distance between the CARbox and the *AOX2* coding sequence, plus the >4 kb non-protein-coding sequence between *AOX2* and the most proximal upstream gene, results in a much longer than average (557 bp) intergenic region for *C. albicans*^53^. In this polymorphic fungus, genes encoding key transcription factors involved in morphogenesis and epigenetic switching between cell types are typically preceded by long 5’ intergenic regions (>7 kb) where multiple regulators can be recruited through protein-DNA interactions and protein-protein condensates to assemble highly complex transcriptional regulatory networks^54^. Given the unusually long intergenic region present, coupled with the complex regulation we uncovered, it seems possible that such super-enhancer-like mechanisms could be involved in coordinating the function of the TFs we have identified binding at the *AOX2* promoter.

From an organismal perspective, the complexity and magnitude of the transcriptional control we uncovered for *AOX2* highlights the importance of Aox activity in enabling *C. albicans* virulence. Our findings begin to reveal how integration of diverse respiratory-stress stimuli can be achieved at the transcriptional level to enable adaptation of this opportunistic fungus as it shifts from harmless commensal to invasive pathogen. The insights gained deepen our molecular understanding of fungal pathogenesis, open new avenues for investigation, and could have important implications for Aox2 function as a promising, but yet to be exploited, target for antifungal drug development^16,55,56^.

## Methods

### Ethics statement

All procedures involving animals were performed under Protocol AN189431-02C, which complies with the National Institutes of Health Guidelines for the Ethical Treatment of Animals and was approved by UCSF’s Institutional Animal Care and Use Committee.

### Fungal strains and culture conditions

All strains were archived in 25% glycerol at −80 °C for long-term storage and freshly streaked on YPD agar medium within 1-week prior to experiments. Strains grown on agar plates were maintained at ambient temperature without refrigeration. In this study, YP-based media contained 1% yeast extract, 2% peptone, 0.2 mM uridine, and one of the following carbon sources: 2% glucose (YPD), 2% galactose (YPGal) or 3% (v/v) glycerol (YPGly). One liter of synthetic complete medium (SC) contained 6.7 g yeast nitrogen base (YNB; with ammonium sulfate), 2 g Drop-out mix without uracil, 0.2 mmol uridine, and one of the following carbon sources: 20 g glucose, 20 g galactose, 30 mL glycerol, 20 g sodium acetate, 20 g casamino acid, 20 g sodium citrate or 30 g sodium lactate. One liter of low-copper SC medium contained 1.7 g YNB (without ammonium sulfate, ferric chloride, or copper sulfate; US Biologicals), 1 g monosodium glutamate hydrate, 2 g Drop-out mix without uracil, 0.2 mmol uridine, 20 g galactose, and 2.5 µmol ferric chloride. To induce copper starvation, strains were grown to saturation in low-copper SC medium and diluted into fresh low-copper medium supplemented with 50 µM bathocuproinedisulfonic acid (BCS) for ~5–6 h. Sulfite treatment was performed in acidified YPGal (adjusted to pH 4.0 with HCl). Cells were grown in buffered YPGal (adjusted to pH 7.2 by the addition of 25 mM HEPES-KOH) before treatment with sodium nitroprusside or DPTA NONOate. Solid media contain 1.5–2% agar.

### Systemic candidiasis model in mice

Experiments were performed with 8- to 10-week-old female BALB/c mice (*n* = 5 or *n* = 8 per experimental group) from Charles River Laboratories (Strain code 028). Systemic infection with *C. albicans* strains was performed by injection of 1 × 10^5^ Colony-Forming Units (CFU) of mid-log-phase yeast into the retrobulbar sinus under isoflurane anesthesia. Animals were provided with fresh water and chow ad libitum, Housing was performed under a 12:12 light: dark cycle in a well-ventilated room (10-15 air changes per hour) with stabilized temperature (68–79 degrees Fahrenheit) and humidity (30-70%). Infected animals were assessed at least once a day for signs of significant morbidity (decreased motor activity, respiratory distress, or a body condition score (BCS) of 2 or less). To minimize suffering and guarantee humane euthanasia, animals that displayed any of these criteria were immediately euthanized by two independent procedures (administration of CO2, followed by cervical dislocation or bilateral thoracotomy).

### Determination of fungal burden in organs

Mice (*n* = 5) were infected with 1 × 10^5^ CFU with the *aox2*-deletion mutant or the complemented strain via the retrobulbar sinus. The kidney, liver, spleen, and brain were collected on Day 3 post-infection. Organs were weighted and homogenized in 1 mL of saline solution (0.9%). Serial dilutions were performed and plated on Sabouraud agar. CFUs were counted after 2 days of growth at 30 °C and were normalized by the organ’s weight.

### Histological analysis of infected kidneys

Kidneys from mice infected with *aox2*-deletion mutant or the complemented strain were collected on Day 3 post-infection and formalin-fixed/paraffin-embedded using standard methods. Serial sections (4 µm) taken at three non-contiguous levels (>70 µm between levels) were stained by routine H&E and PAS methods. For IHC, α-Candida (1:1000, Abcam ab53891) and α-CD45 (1:1000, Invitrogen RA3-6B2) antibodies were used followed by detection with diaminobenzidine (DAB) according to manufacturer’s recommendations (ImmPACT DAB Substrate Kit, Peroxidase-Biolynx, Vector Laboratories, SK-4105). Slides were imaged on a 3DH Pannoramic Slide Scanner at 40X magnification (bright field).

### Commensalism model in mice

The mouse model of *C. albicans* commensalism was performed as previously described^57,58^. Female BALB/c mice (*n* = 4, 8–10-week-olds) were singly housed and treated with penicillin 1500 un/ml and streptomycin 2 mg/mL in drinking water starting 7 days before gavage and continuously for the remainder of the experiment. Feces were plated on Sabouraud agar, and LB agar to monitor for fungal and bacterial growth. Mice were infected with 10^8^ CFUs of a 1:1 mixture of the *aox2*-deletion mutant and the complemented strain in 0.9% saline by gavage. *C. albicans* was recovered from the inoculum and host feces every 5 days for 3 weeks, and strain quantification was performed by qPCR using primers listed in Supplementary Data 2.

### Strain construction

Detailed information regarding the strains used in this study, including names, genotypes, and figure panel(s) in which each strain was presented are provided in Supplementary Data 1. The transient CRISPR system developed by Min et al. ^59^. was used to tag or delete both alleles of *C. albicans* genes of interest. The tagging system described by Zhang et al. ^60^. was used to modify *C. albicans* to express proteins with C-terminal tags from their endogenous promoters. Additional details regarding strain and plasmid construction are available upon request.

### Dose-response and spotting growth inhibition assays

Strains grown overnight in YPD were diluted to ~50,000 cells/mL in the appropriate medium. 100 µL (or 20 µL) of the diluted culture was mixed with an equal volume of medium supplemented with a given inhibitor at the specified range of concentrations in 96- or a 384-well plate format. Growth (OD_600_) in SC-glucose or SC-galactose was measured after incubation at 30 °C for 48 h. Ten-fold higher concentration inocula were used when glycerol, acetate, lactate, or citrate was the carbon source and growth was measured after 72-hour incubation. Unless otherwise specified, growth (OD_600_) in the presence of inhibitor was normalized to growth in a compound-free medium. All dose-response assays were performed at least twice with comparable results. The mean of the technical duplicates from one representative experiment was presented in heatmap format visualized by Java TreeView, version 1.1.6r4 (http://jtreeview.sourceforge.net). For spotting growth assays on agar medium, strains were grown to saturation in tubes under shaking conditions or statically in a 96-well plate. Ten-fold serial dilutions of cultures were made in water and spotted onto agar plates using a multi-channel pipettor or a 96-well replicator.

### Compound treatments

The growth medium, compound concentration, and duration of each treatment are specified for each experiment in the Figure Legend. Strains were grown overnight in the same medium in which treatment would be performed. The saturated culture was diluted in fresh medium (1: 50 for YPGly, 1:100 for YPGal, and 1:200 for YPD) and sub-cultured for ~4–5 h before treatment. Antimycin A, rotenone, myxothiazol, and Inz-5 were added to the indicated final concentration from 10 mM DSMO stocks stored at −20 °C. KCN solution (1 M) was kept in dark at room temperature. Fresh stocks were prepared every 2-3 months. Menadione in DMSO (0.25 M) and sodium sulfite in water (0.1 M) were freshly prepared for each experiment. Sodium nitroprusside dihydrate (0.5 M in water) and DPTA NONOate (0.1 M in YPGal medium buffered to pH 7.2 by 25 mM HEPES-KOH) were prepared ~10 min before being added to fungal cultures.

### Quantitative reverse transcription PCR

Total RNA preparation and reverse transcription were performed as previously described^61^. Reactions containing cDNA reverse-transcribed from 1 µg RNA were 1:80 diluted in water and then diluted samples (4 µL) were used to assess relative gene expression in a 10 µL SYBR green quantitative PCR reaction (Fast SYBR™ Green Master Mix) with *ACT1* level as the internal control. Sequences of the qPCR primers used in this study are listed in Supplementary Data 2.

### β-galactosidase activity assays

Strains carrying each LacZ reporter construct were grown to early-log-phase in YPGly medium and aliquoted into a 96-well plate (150 µL/well). After the addition of 10 µM AA (or DMSO vehicle control), cells were grown for ~4 h at 30 °C under shaking conditions. β-galactosidase activity was then measured using a Gal-Screen^TM^ kit by mixing an equal volume of cell culture (30 µL) and Buffer B (provided in the kit) in a 384-well plate. Luminescent signals were recorded by a Tecan plate reader (controlled by Magellan 7.2) every 15 min for 90 min during incubation at 30 °C and peak signal, which was typically reached after 60-75 min, was used to compare induction of *LacZ* after normalization to the OD_600_ of the input culture.

### Purification of recombinant Rtg1/Rtg3-6Xhis complex from *E. coli*

Codon-optimized sequences were inserted between the NcoI/SacI (Rtg1) and the NdeI/XhoI (Rtg3-6XHis) sites of the pColaDuet vector (Novagen) for co-expression in *E. coli* (Supplementary Data 3). A BL21(DE3) expression strain carrying the pColaDuet-Rtg1-Rtg3X6his plasmid (pLC1452) was grown in four liters of LB+Kan liquid medium (1% Tryptone, 0.5% yeast extract, 1% NaCl and 50 µg/mL kanamycin) to OD_600_ ~ 0.6 at 37 °C. Expression of the recombinant protein was induced for ~4 h at 30 °C after addition of 1 mM IPTG. Cells were collected by centrifugation at 12,000 × *g* for 15 min and resuspended in 35 mL (per liter culture) lysis buffer (50 mM NaPi, pH 8.0, 300 mM NaCl and 5 mM imidazole) and sonicated for 20 min (10 sec on; 10 sec off) at 30% amplitude using a Misonix S-4000 dual horn sonicator with 3/4-inch probes. Lysates were then clarified by centrifugation at 35,000 × *g* for 25 min. The supernatant was filtered through 0.45 µm PVDF membrane and incubated with Ni-NTA agarose (2 mL bed volume) for 1 h. Beads were collected into a chromatography column and washed with 40 mL of wash buffer (50 mM NaPi pH 8.0, 600 mM NaCl, and 15 mM imidazole). Bound proteins were eluted twice with 2.5 mL elution buffer (50 mM NaPi pH 8.0, 300 mM NaCl, and 250 mM imidazole). After buffer-exchange on a PD-10 desalting column to T150 buffer (25 mM Tris-HCl pH 7.5, 150 mM NaCl, and 5% glycerol), the product was applied to HiTrap Heparin HP (5 mL; GE) and eluted by increasing concentrations of NaCl (0.15 to 1 M). Peak fractions were combined and re-concentrated on ~0.6 mL bed volume of Ni-NTA agarose. This sample was resolved on HiLoad 16/600 Superdex 200 pg size-exclusion column (SEC; running buffer: 25 mM HEPES-NaOH pH 7.8, 300 mM NaCl, and 5% glycerol). All centrifugation and Ni-NTA purification steps were performed at 4 °C, while heparin and size-exclusion chromatography were performed at ambient temperature. All buffers were supplemented with a protease inhibitor cocktail immediately prior to use (1 mM PMSF, 2 mM benzamidine HCl, 0.6 μM leupeptin, and 2 μM pepstatin A).

### Gel-shift assays

The concentration of recombinant Rtg1/Rtg3-6XHis in peak SEC elution factions was estimated using a molar extinction coefficient of 47,220 M^−1^ cm^−1^ at 280 nm. Serial dilution of the recombinant proteins (Mix A) in SEC running buffer was made from an ~7.5 μM fraction. DNA probes were amplified from plasmids encoding WT or mutant *AOX2* promoters using ExTaq polymerase, purified on QuickSpin columns, and stored as 20 nM stocks in EB buffer. The TCA triplet closely following the GTCA motif 1 or motif 3 was disrupted with the associated GTCA motif to generate mutant vectors and probes. Sequences of the probes and the full-length *AOX2(A)*/*(B)* promoters cloned from wild-type strain SN95 are provided in Supplementary Data 2 and 3, respectively. Mix B was made by diluting the probes to 2.67 nM in a buffer containing 25 mM HEPE-NaOH pH 7.2, 1 mM EDTA, and 5% glycerol. 5 µL of Mix A and 15 µL of Mix B were combined in a low-binding 1.5 mL tube (VWR) and incubated for ~15 min at ambient temperature. Samples were directly loaded on a 5% TBE-glycerol acrylamide gel (5% acrylamide/bis-acrylamide (29:1), 0.5× TBE and 2.5% glycerol), which had been pre-run for ~30 min at 100 V. After electrophoresis at 10 mA for ~80 min in 0.5XTBE + 2.5% glycerol at ambient temperature, gels were stained in running buffer containing 1× SYBR Green for 20 min and imaged using ChemiDoc (Bio-Rad) after three rinses in distilled water.

### Immunoprecipitation

Strains, growth media, and compound treatment for IP experiments are specified in each Figure Legend. Routinely, cultures (100 mL) in YPGal before and after AA or KCN treatment were harvested by centrifugation at 3000 × *g* for 2 min in 50 mL conical tubes. Cell pellets were washed in 1 mL of F300 buffer (50 mM HEPES-KOH pH 7.2, 300 mM KOAc, and 10% glycerol) and stored at −80 °C. To prepare whole cell lysate, each pellet was resuspended in 1 mL F300 and disrupted by bead-beating (2 min on; 1 min rest on ice) for three cycles. Crude lysates were mixed with a half-volume of F300 supplemented with 0.06% IGPAL-CA630 and sonicated (20 sec × 4) at 30% amplitude using a 0.63-cm diameter probe. Sonicated lysates were treated with ~30 U Benzonase (Millipore) for 20 min after the addition of MgCl_2_ to the final centration of 2 mM and clarified by centrifugation at 20,000 × *g* for 20 min. Total protein (~6 mg) was incubated with ~6 µg of anti-Flag (or anti-HA) antibody for 90 min on a revolving rotator. Protein G Dynabeads (40 µL) were added to each IP and incubated for 90 min. Bead-bound antibody-protein complexes were captured using a magnetic rack and washed with F300 + 0.02% IGEPAL-CA630 × 3 before boiling in 1× SDS-PAGE loading buffer (40 µL). Proteins were resolved on 10% precast SDS-gels and blots probed with HRP-conjugated anti-Flag antibody (A8592; Sigma; 1:3000) or anti-HA antibody (3F10; Roche; 1:3000) followed by HRP-conjugated goat anti-Rat secondary antibody (A10549 Invitrogen; 1:7000). All F300 buffers were supplemented immediately prior to use with 1× PhosSTOP (Roche) and 1× Complete inhibitor cocktails (Roche) to inhibit phosphatase and proteinase activity.

### Whole-cell lysate preparation under denaturing conditions and immunoblotting

Whole cells lysates for assessing relative protein level or gel mobility were prepared using a TCA-precipitation-based method as previously described with slight modification^62^. Precipitated protein pellets were rinsed in cold acetone and resuspended in a modified loading buffer (40 mM Tris-HCl pH 6.8, 8 M urea, 5% SDS, 0.1 M EDTA), which did not contain β-mercaptoethanol or bromophenol blue. Samples were diluted 1:20 in 1% SDS to measure protein concentration by DC protein assay kit using BSA as a standard. Samples were diluted to the same protein concentration and mixed with 1/5 volume of the modified loading buffer supplemented with 0.6% (v/v) β-mercaptoethanol and ~0.3 mg/mL bromophenol blue. Total protein (~30 µg) in samples was resolved on 10% SDS-gels and blots probed using anti-Flag (F3165; Sigma; 1:3000) or anti-HA (3F10; Roche; 1:3000) primary antibody and the corresponding HRP-conjugated secondary antibody. Blots were developed using Clarity^TM^ ECL substrate (Bio-Rad) and chemiluminescent signals captured by ChemiDoc Imager (Bio-Rad).

### Chromatin immunoprecipitation assay (ChIP)

ChIP assays were performed as previously described with minor modifications^62^. Cells were fixed in 1% formaldehyde for 20 min at room temperature with agitation. Crude lysates recovered after bead-beating were successively sonicated using a probe (0.63-cm diameter; 20 sec × 4; 30% amplitude) and then a water bath sonicator (Bio-Disrupter; High setting; 5 min × 4; 30 sec on/30 sec off). Antibodies used for anti-Flag and anti-HA ChIP were F3165 (Sigma) and sc-7392 X (Santa Cruz) respectively. The abundance of the DNA fragments of interest in input samples and IP products were assessed by qPCR using primers listed in Supplementary Data 2.

### Fluorescence microscopy

Strains and growth conditions for each microscopy experiment are specified in Figure Legends. Routinely, ~ 1 mL of log-phase cells was pelleted by centrifugation. After washing in 500 µL PBS twice, cells were resuspended in ~150 µL PBS and immediately imaged under a Zeiss Axio Imager MI microscope (Carl Zeiss) with an X-cite series 120 light source for fluorescence. Images were captured and processed by the ZEN pro program (Zeiss).

### Statistics and reproducibility

All RT-qPCR, ChIP-qPCR experiments, and dose-response assays were performed at least twice and/or using independent transformants to ensure reproducibility. Except where indicated in specific figure legends, data are presented as the mean (SD) of technical replicates from one representative biological experiment whenever major (>5-fold differences between test and control groups) were observed. Data for the corresponding biological replicate confirming reproducibility are provided in the source data file. When less than twofold differences between test and control groups were observed, data from three biological replicates are presented and significance was determined by unpaired, two-tailed Student’s *t* test. GraphPad Prism (7.04) was used to perform Kaplan–Meier survival analysis, generate graphical displays, and perform tests of significance using Log-rank (Mantel-Cox) tests. Microscopic analyses of fungal morphology (Supplementary Fig. 1c) or localization of GFP/RFP fusion proteins (Figs. 2f, 4i, 5d; Supplementary Figs. 4e, f, 5g, i) were performed twice using biologically distinct samples to ensure reproducibility. Representative fields containing multiple (≥4) cells from one experiment were presented. Results of gel-shift assays (Fig. 4c), immunoprecipitation (Fig. 5c; Supplementary Fig. 5k, 6j), and immunoblotting comparing protein abundance or mobility (Figs. 2e, 5g, 6f; Supplementary Fig. 3c, 5e, h, j, 6d) were representative of two biological replicates. For purification of the recombinant Rtg1/Rtg3-6XHis complex (Fig. 4b), multiple batches of Ni-NTA products showed a similar elution profile from the Heparin column. Size-exclusion chromatography was performed once using pooled Heparin eluates.

### Reporting summary

Further information on research design is available in the Nature Portfolio Reporting Summary linked to this article.

## Supplementary information

Supplementary Information

Description of Additional Supplementary Files

Supplementary Data 1

Supplementary Data 2

Supplementary Data 3

Reporting Summary

## Data Availability

Source data are provided in this paper, which includes all raw data reported within this manuscript. Source data are provided in this paper.

## Source data

Source Data
