# Supplementary material for: Multifactor transcriptional control of alternative oxidase induction integrates diverse environmental inputs to enable fungal virulence
Source: Nat Commun. 2023 Jul 27;14:4528. doi: 10.1038/s41467-023-40209-w (PMC10374912; doi:10.1038/s41467-023-40209-w)
Supplement: Supplementary file 1 — Supplementary Information [file 41467_2023_40209_MOESM1_ESM.pdf]

Supplementary Information

Multifactor transcriptional control of alternative oxidase induction integrates diverse environmental inputs to enable fungal virulence

Zhongle Liu, Pauline Basso, Saif Hossain, Sean D. Liston, Nicole Robbins, Luke Whitesell, Suzanne M. Noble, and Leah E. Cowen

Supplementary Figure 1

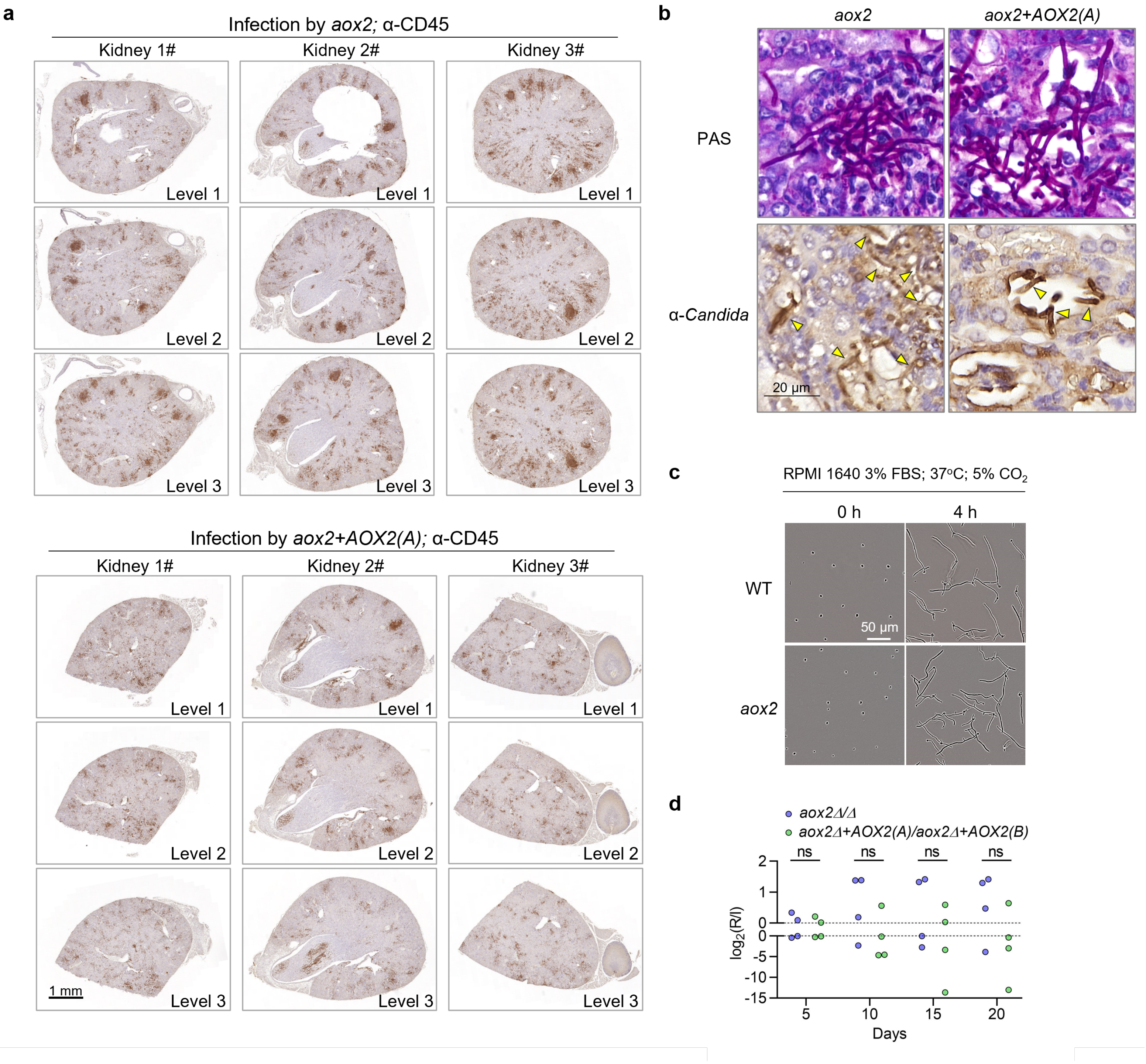

Supplementary Figure 1: Deletion of *AOX2* increases leukocyte infiltration of infected kidneys during early systemic infection without affecting fungal morphogenesis or fitness in a gut commensalism model. a α-CD45 IHC staining of kidneys from mice (n=3) systemically infected by the *aox2*-deletion mutant

or the add-back strain. Staining of sections from three non-contiguous levels are shown for each kidney. **b** *AOX2* is not required for filamentation *in vivo.* Sections of kidneys infected with the *aox2*-deletion mutant or the add-back strain (see panel **a**) were stained by the PAS method or a polyclonal α-*Candida* antibody. The elongated cell structures highlighted in magenta (PAS) or in brown (α-*Candida*; indicated by yellow arrowheads) represent *C. albicans* filaments and were observed in multiple sections. Representative fields were shown. **c** *AOX2* is not required for *in vitro* hyphal growth under tissue culture conditions. WT *C. albicans* and the *aox2*-deletion mutant were incubated for four hours in FBS-supplemented RPMI 1640 medium at 37 °C under 5% CO_2. Cell morphology was assessed by microscopy before and after incubation. **d** *AOX2* is dispensable for *C. albicans* to colonize the gut of mice in a competition commensalism model. The *aox2* mutant and an add-back strain (with both *AOX2* alleles re-introduced) were co-inoculated into mice by gavage (n=4) at an ~1:1 ratio. Changes in the population ratio of the two strains in samples of feces were tracked by qPCR analysis of extracted fungal genomic DNA. ns: not significant by two-tailed Mann-Whitney test (p>0.05).

Supplementary Figure 2

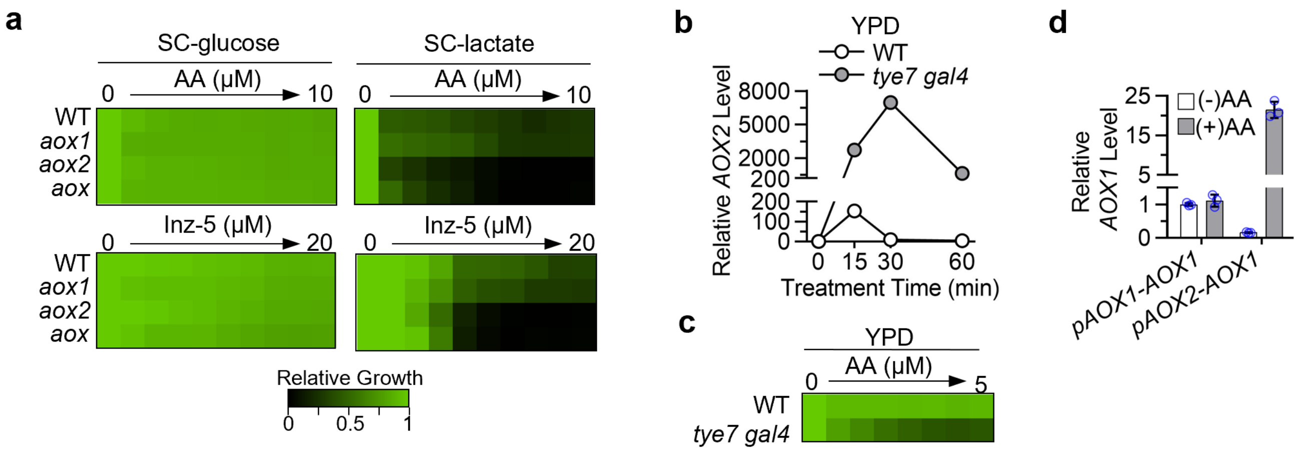

Supplementary Figure 2: Transcriptional response of *AOX2* to Complex III inhibition is regulated by the carbon source and glycolytic flux. a Growth of *aox2*-null mutant is hypersensitive to Complex III inhibition in lactate-, but not glucose-supplemented medium. WT *C. albicans* and mutants with deletion of the *AOX* genes were tested for growth inhibition by AA and Inz-5 in SC-glucose (fermentable) or SC-lactate (non-fermentable). **b** Compromising glycolysis increases the AA-inducibility of *AOX2* in glucose-containing medium. WT and *tye7/gal4*-deletion strains grown in YPD were treated with AA (10 μM). *AOX2* transcript levels were measured at the indicated intervals. Basal *AOX2* level in the WT strain was used to normalize relative expression under all other conditions. **c** Concentration-dependent growth inhibition of the *tye7/gal4*-deletionmutant by AA in glucose-containing medium (YPD). **d** *AOX1* expression under control of the *AOX2*, but not *AOX1* promoter, is AA-inducible. *AOX1* was placed under control of *AOX1* or *AOX2* promoter elements in an *aox*-deletion background and strains grown in YPGly supplemented with AA (10 μM) for 60 minutes. *AOX1* transcript levels before and after AA addition were measured by RT-qPCR. Basal *AOX1* expression from the native promoter was used to normalize relative expression under all other conditions. Panels **b** and **d** present the mean (SD) of technical triplicates from an experiment representative of biological duplicates with comparable results. See source data file for results of the biological duplicate.

Supplementary Figure 3

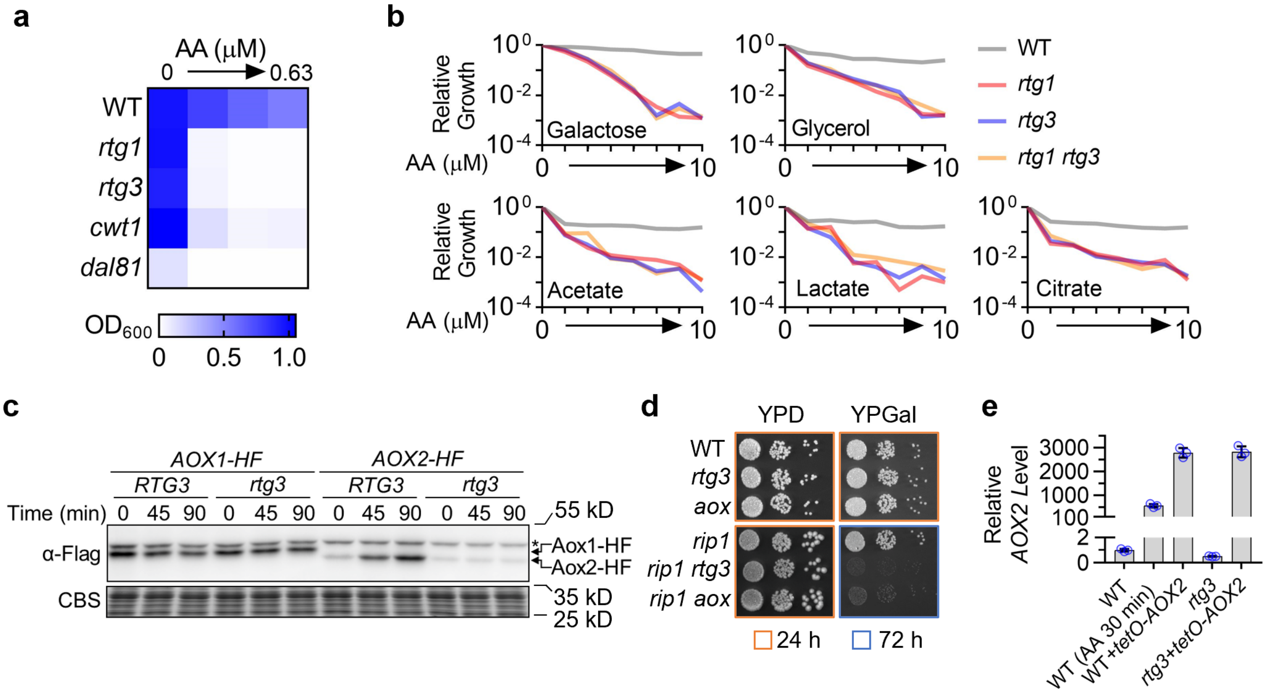

Supplementary Figure 3: Rtg1/Rtg3 function is required to utilize diverse alternative carbon sources upon inhibition of the classical ETC. **a** Heat-map depiction comparing growth inhibition by AA (OD_{600} at 48-hr) of the hit strains identified by the screen presented in Fig. 3A to growth inhibition of the WT reference strain. **b** Relative growth inhibition caused by a 2-fold dilution gradient of AA was monitored using OD_{600} as endpoint for parental WT strain and mutants with deletion in *RTG1*, *RTG3* or both genes (see **Fig. 3b**). Strains were cultured in SC media supplemented with the indicated carbon sources. For each strain, ‘relative growth’ was normalized to growth in the relevant AA-free medium and plotted on a $\log_{10}$ *y*-axis. Lower limit of detection for the assay was ~10^{-3}. To allow for direct comparison, data presented in **Fig. 3a** for galactose- and glycerol-containing media were re-plotted in the same format used for the additional carbon sources presented here. **c** Deletion of *RTG3* abolishes the increase in Aox2 protein levels induced by AA. WT and *rtg3*-deletion strains that express C-terminal 6XHis-3XFlag (HF) tagged Aox1 or Aox2 from the native promoters were treated with AA (10 μM) in YPGly medium for varying intervals prior to lysis. Whole cell lysates were resolved by SDS-PAGE and blots probed with anti-Flag antibody. Asterisk denotes a non-specific band. **d** Deleting *RTG3* and *RIP1* in combination abolishes growth on YPGal agar. Strains with deletion(s) in the indicated gene(s) were

spotted on YPD or YPGal agar. Images were obtained after 24-hr or 72-hr incubation at 30°C. **e** Confirmation of *RTG3*-independent over-expression of *AOX2* from a heterologous promoter. Relative *AOX2* expression driven by its native promoter or a strong *tetO* promoter was compared in WT and *rtg3*-deletionbackgrounds in SC-galactose medium. As a benchmark, relative *AOX2* expression was also measured in the WT strain after AA treatment (10 μM for 30 min). Data represent the mean (SD) of technical triplicates from an experiment representative of biological duplicates with comparable results. See source data file for results of the biological duplicate.

Supplementary Figure 4

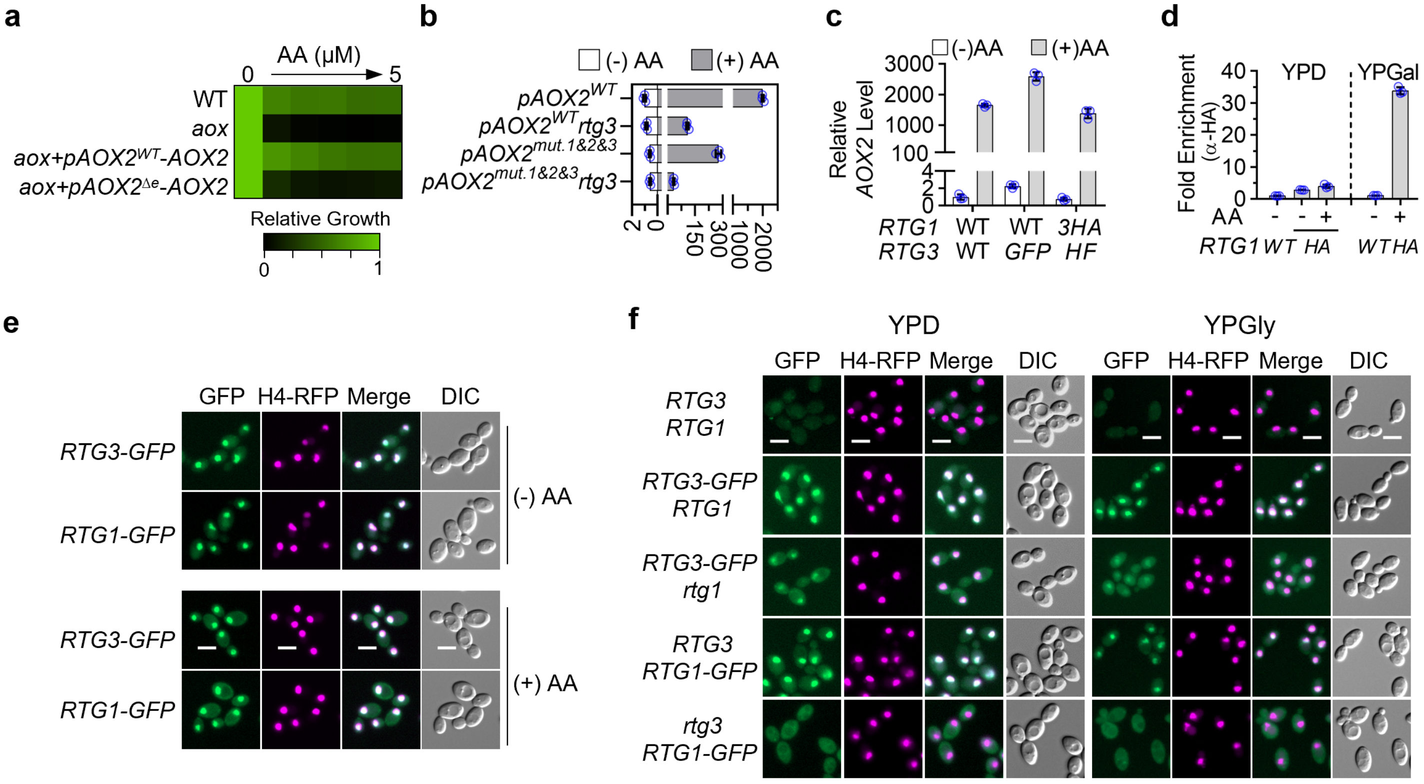

Supplementary Figure 4: The CARbox is the primary promoter element mediating *AOX2* transactivation by antimycin A through interactions with the Rtg1/Rtg3 complex*.* **a** A strain expressing *AOX2* from a CARbox-deleted promoter is hypersensitive to AA. *AOX2* driven by a WT or mutant (deletion of nucleotides -1270 to -1039*)* promoter was re-introduced in an *aox*-deletion background and strains tested for relative growth inhibition by AA in SC-galactose. The WT and parental *aox*-deletion strains served as controls. Growth was quantified and visualized as described in **Fig. 2c** (see colour bar). **b** Relative *AOX2* expression driven by either a WT or mutant *AOX2* promoter with mutations in all three GTCA motifs was measured by RT-qPCR in WT and *rtg3*-deletion backgrounds with or without AA treatment (10 µM for 30 min) in YPGal. Data for the corresponding biological replicate confirming reproducibility are provided in the source data file. **c** C-terminal tagging does not affect function of the Rtg1/Rtg3 complex in transactivating *AOX2* upon AA treatment. Strains that express C-terminal GFP-tagged Rtg3 ('GFP') or co-express 6XHis-3XFlag-tagged (HF) Rtg3 and 3XHA-tagged Rtg1 as the sole source for each protein were grown in YPGly and treated with AA (10 µM for 1 h). **d**

AA treatment in YPD does not induce Rtg1 binding to the *AOX2* promoter. Rtg1 binding at the CARbox was measured by anti-HA ChIP in the Rtg1-3HA-expressing strain ('HA') before and after AA treatment (10 μM for 15 min) in either YPD or YPGal. The level of background signal was determined using an untagged strain ('WT'). **e** Rtg1-GFP and Rtg3-GFP signals localize to the nucleus under both non-inducing and AA-inducing conditions. Cells modified to express Rtg3-GFP (or Rtg1-GFP) and RFP-tagged histone H4 (nuclear marker) were grown in YPGal and imaged by fluorescence microscopy before and after AA treatment (10 μM for 20 min). Scale bar: 5 μm. **f** Localization of Rtg1-GFP and Rtg3-GFP when glucose or glycerol is the medium's carbon source. The fluorescence microscopy images were obtained using the strains presented in **Fig. 4i** following growth in YPD or YPGly medium. Scale bar: 5 μm. Panels **b-d** present the mean (SD) of technical triplicates from an experiment representative of biological duplicates with comparable results. See source data file for results of the biological duplicate.

Supplementary Figure 5

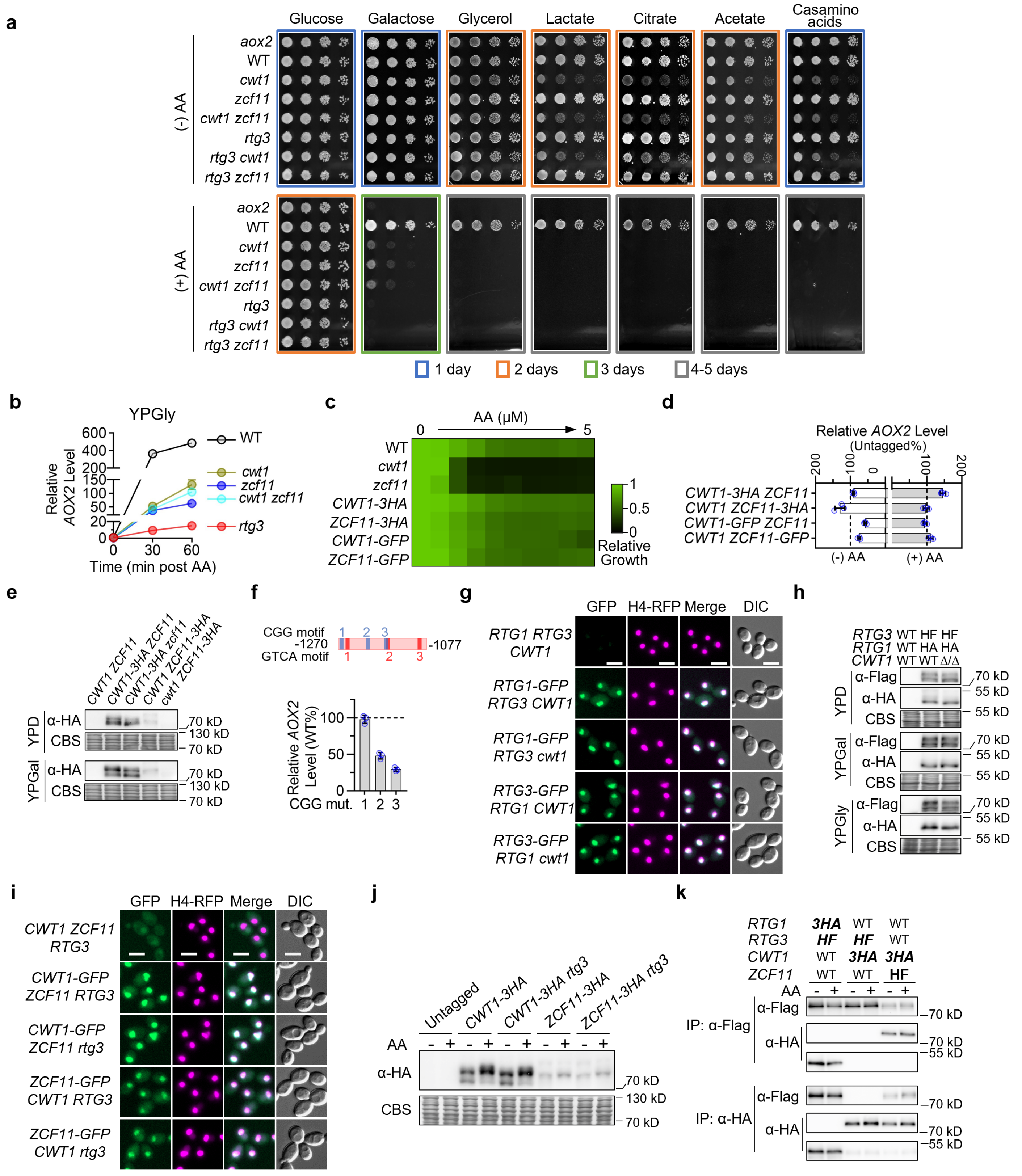

Supplementary Figure 5: The Cwt1/Zcf11 complex is not required for expression or localization of the Rtg1/Rtg3 complex and *vice versa.* **a** Strains with deletion of *CWT1* or *ZCF11* are hypersensitive to Complex III inhibition on solid media containing various non-fermentable carbon sources. The transcription factor mutants characterized in **Fig. 5a** were tested for sensitivity to growth inhibition by AA (2 μM) on SC-agar containing the indicated carbon sources. The WT parent and the *aox2*-deletion mutant were included as controls. The incubation time for each plate is color-coded by the plate outline. **b** Deletion of *CWT1* or *ZCF11* decreases *AOX2* induction by AA in YPGly. Mutants with individual or combined deletion of *CWT1* and *ZCF11* were treated with AA (10 μM) in YPGly. *AOX2* expression levels in mutants were compared with levels in the WT and the *rtg3*-deletion strains. **c-d** Function of Cwt1 and Zcf11 in transactivating *AOX2* is unaffected by C-terminal tagging. Strains engineered to encode Cwt1 or Zcf1 tagged with C-terminal 3XHA or GFP under control of the endogenous promoters for both alleles of each gene showed WT-like sensitivity to growth inhibition by AA in SC-galactose (**c**) and WT-level of *AOX2* expression before and after AA treatment in YPGal (**d**). *AOX2* transcripts levels in the tagged strains are presented as percentage of the level in the untagged parent strain in **d**. **e** Zcf11 is the less abundant component of the Cwt1/Zcf11 complex and its level diminished by deletion of *CWT1*. Whole cell lysates prepared from WT and *zcf11*-deletion (or *cwt1*-deletion) strains that express Cwt1-3XHA (or Zcf11-3HA) from their endogenous promoters were resolved by SDS-PAGE and the blots probed by anti-HA antibody. Lysates made from the untagged strain grown in YPD and YPGal were included to confirm specificity of immunoblotting signals. **f** Identification of CGG motifs important for CARbox function. *Top*: Schematic view of the CGG motifs within CARbox, numbered 1 to 3 (blue font) from the 5' to the 3' end. The location of GTCA motifs bound by the Rtg1/Rtg3 complex are shown in red. *Bottom*: Levels of AA-induced *AOX2* expression from promoters carrying mutations of the CGG motifs are shown as percentage of the level driven by the WT promoter. **g** Deletion of *CWT1* or *ZCF11* does not affect nuclear localization of the Rtg1/Rtg3 complex. Rtg1-GFP and Rtg3-GFP expressed from the endogenous promoters co-localized with the H4-RFP nuclear marker in WT, *cwt1*-deletion, and *zcf11*-deletion strains grown in YPGal. Scale bar: 5 μm. **h** Deletion of the Cwt1/Zcf11 complex does not change protein levels of Rtg1 or Rtg3. Rtg1-3XHA and Rtg3-6XHis-3XFlag (HF) were expressed from their native promoters in WT and *cwt1*-deletion strains, and protein levels were compared

after growth in YPD, YPGal, and YPGly. **i** Deletion of the Rtg1/Rtg3 complex does not alter localization, expression, or gel mobility of Cwt1 or Zcf11. Fluorescence microscopy images show co-localization of Cwt1-GFP and Zcf11-GFP with the H4-RFP nuclear marker in both WT and *rtg3*-deletion backgrounds. **j** Anti-HA immunoblotting compares relative levels and gel mobility of Cwt1-3XHA and Zcf11-3XHA in the *rtg3*-deletion strain with those in the WT strain. Cultures analyzed in **i** and **j** were grown in YPGal and treated with AA (10 μM) for 15 min as indicated. **k** The Cwt1/Zcf11 complex and the Rtg1/Rtg3 complex do not co-immunoprecipitated as a 'super' complex. α-HA and α-Flag immunoprecipitation assays were performed to detect potential inter-complex interaction in the strain that expresses 3xHA-tagged Cwt1(-3XHA)/Zcf11 complex and 6XHis-3XFlag tagged Rtg1/Rtg3-(HF) complex. Lysates were prepared from cells grown in YPGal with and without AA treatment (10 μM for 15 min). Panels **b**, **d** and **f** present the mean (SD) of technical triplicates from an experiment representative of biological duplicates with comparable results. See source data file for results of the biological duplicate.

Supplementary Figure 6

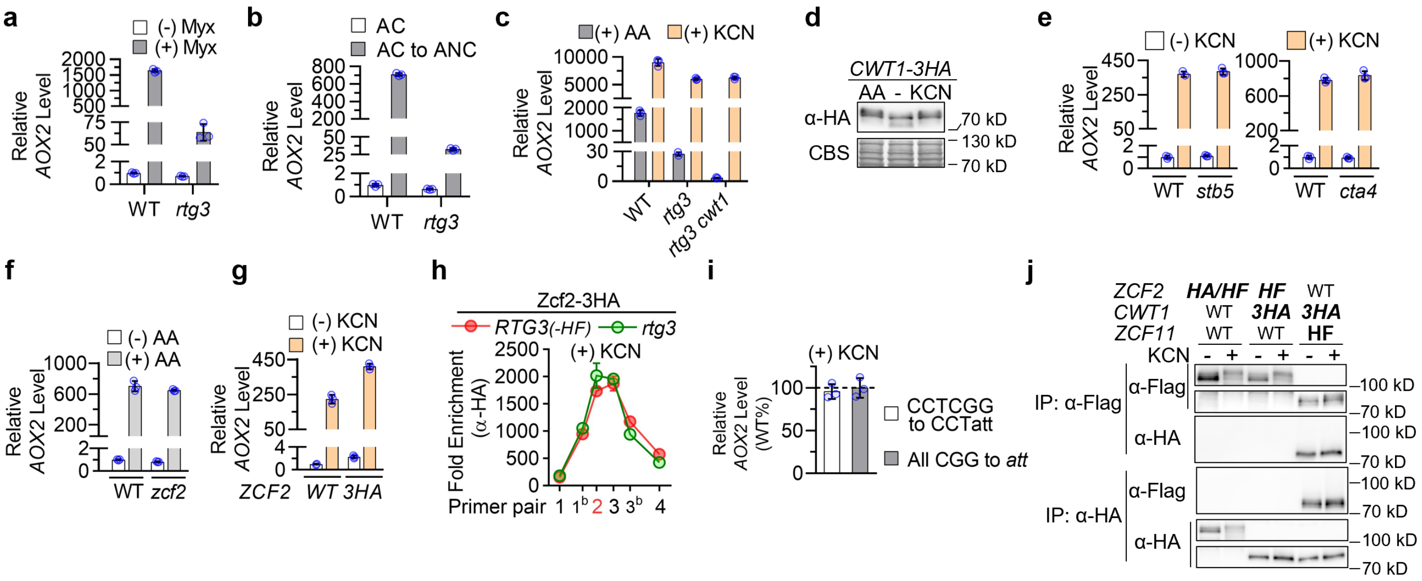

Supplementary Figure 6: **Cyanide induces *AOX2* expression through two mechanistically distinct pathways**. **a** *RTG3*-dependent transactivation of *AOX2* by myxothiazol treatment. *AOX2* expression levels in WT and *rtg3*-deletion strains were assessed before and after myxothiazol treatment (10 μM for 20 min) in YPGal. **b** Aerobic-to-anaerobic shift induces *AOX2* expression. The WT and *rtg3*-deletion strains growing in YPGal (aerobic; AC) were shifted to an anaerobic chamber (ANC; 10% H_2, 10% CO_2 and 80% N_2) and incubated for 1 hour. *AOX2* transcript levels were measured in cultures with and without the shift. **c** Rtg3-independent *AOX2* transactivation induced by KCN is not mediated by Cwt1. *AOX2* transcripts levels were assessed in the WT, *rtg3*-deletion and *rtg3*/*cwt1*-deletion strains after 20-min treatment with AA (10 μM) or KCN (1 mM) in YPGal and normalized to the basal *AOX2* level in the WT strain. **d** KCN treatment triggers a mobility shift of Cwt1 in a similar manner to AA treatment. Cwt1 mobility before and after AA (10 μM) or KCN (1 mM) treatment were compared by α-HA immunoblotting using lysates prepared from a Cwt1-3XHA-expressing strain. **e** Zinc-cluster transcription factors Cta4 and Stb5 are not required for *AOX2* induction by cyanide. *AOX2* expression levels in *cta4*- and *stb5*-deletion mutants were compared with that in the parental WT strain before and after cyanide treatment (1 mM for 20 min) in YPD. **f** WT and *zcf2*-deletion strains show comparable *AOX2* expression before and after AA treatment (10 μM for 20 min) in YPGal. **g** C-terminal tagging does not impair the function of Zcf2 in mediating *AOX2* transactivation in

response to cyanide. The strain that expresses C-terminal 3XHA tagged Zcf2 from both copies of the native promoter was treated with 1 mM KCN for 20 min in YPGal. *AOX2* levels before and after treatment were compared with those in the unmodified WT strain. **h** Deletion of *RTG3* does not affect Zcf2 binding to the *AOX2* promoter. Binding profile of Zcf2 at the *AOX2* promoter following cyanide exposure was also assessed in the *rtg3*-deletion strain in the same experiment presented in **Fig. 6g** and compared with the profile in the WT strain. The ChIP products were analyzed by additional primer pairs 1^b and 3^b to assess relative Zcf2 binding at intermediate regions between primer pairs 1/2 and 3/4 respectively. **i** Mutation of CGG motifs does not affect function of the cyanide-responsive element identified in Fig. 6H. The eleven CGG triplets in both alleles of *AOX2* promoter (positions -1360 to -810) were mutated to *att* in concert. Alternatively, one of the eleven CGG triplets that is encompassed by a longer 'CTTCGG' motif was mutated selectively. Levels of KCN-induced *AOX2* expression from the two mutant promoters are shown as percentage of that from the WT promoter. The experiment was performed in an *rtg3*-deletion background to eliminate activation through the Rtg1/Rtg3 complex. **j** Zcf2 does not homodimerize or interact with the Cwt1/Zcf11 complex in immunoprecipitation assays. Strains were modified to express Cwt1, Zcf11 and Zcf2 differentially labeled with C-terminal 3XHA and 6XHis-3XFlag tags in each indicated combination. Anti-HA and anti-Flag immunoprecipitation assays were performed using the recombinant strains to detect potential interaction involving Zcf2. Co-immunoprecipitation of Cwt1-3XHA and Zcf11-HF serves as a positive control for interaction. All strains were growth in YPGal and treated with 1 mM KCN for 15 min. Panels **a-c** and **e-i** present the mean (SD) of technical triplicates from an experiment representative of biological duplicates with comparable results. See source data file for results of the biological duplicate.

Supplementary Figure 7

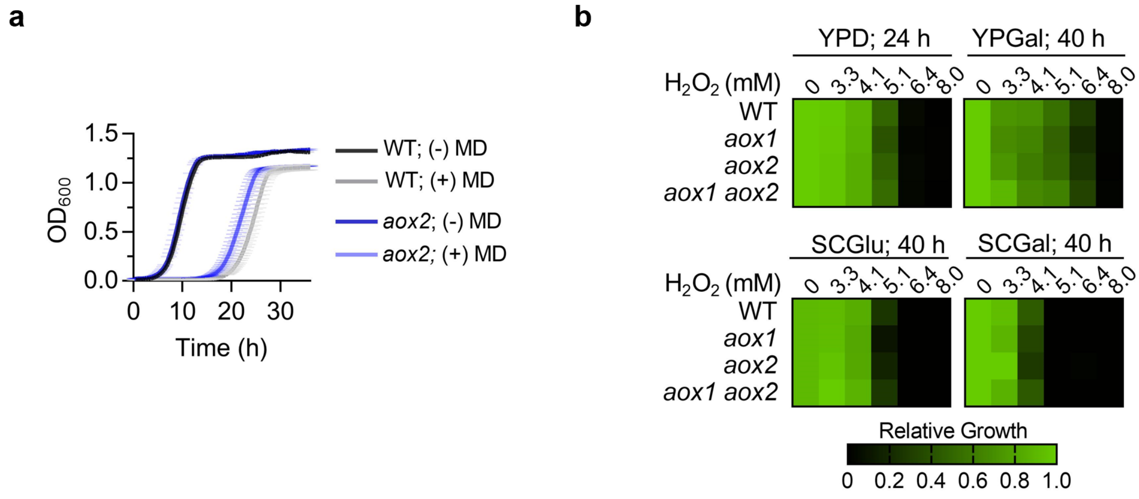

Supplementary Figure 7: *AOX2* does not increase fitness under oxidative stress. a Deletion of *AOX2* does not increase sensitivity to menadione. Growth of WT and *aox2*-deletion mutant in YPD with or without 0.1 mM menadione was monitored by serial measurements of OD_{600}. Data report the mean (SD) of technical triplicates from a representative experiment of biological duplicates with comparable results. **b** Deletion of the alternative oxidase genes does not confer hypersensitivity to hydrogen peroxide. H_2O_2-sensitivity of the *AOX* gene deletion mutants were evaluated in YP and SC media containing glucose or galactose as the carbon source. Relative growth is presented in heat-map format with scale bar.
