# Supplementary material for: Multifactor transcriptional control of alternative oxidase induction integrates diverse environmental inputs to enable fungal virulence
Source: Nat Commun. 2023 Jul 27;14:4528. doi: 10.1038/s41467-023-40209-w (PMC10374912; doi:10.1038/s41467-023-40209-w)
Supplement: Supplementary file 2 — Description of Additional Supplementary Files [file 41467_2023_40209_MOESM2_ESM.pdf]

Description of Additional Supplementary Files

File Name: Supplementary Data 1
Description: Strains used in this study.

File Name: Supplementary Data 2
Description: Sequences of primers and probes used in this study.

File Name: Supplementary Data 3
Description: Sequences of the AOX2 promoters cloned from SN95 and codon optimized RTG1/RTG3 for recombinant expression.
