# Supplementary material for: Multifactor transcriptional control of alternative oxidase induction integrates diverse environmental inputs to enable fungal virulence
Source: Nat Commun. 2023 Jul 27;14:4528. doi: 10.1038/s41467-023-40209-w (PMC10374912; doi:10.1038/s41467-023-40209-w)
Supplement: Supplementary file 6 — Reporting Summary [file 41467_2023_40209_MOESM6_ESM.pdf]

nature portfolio

Corresponding author(s): Leah E. Cowen
Last updated by author(s): Jun 28, 2023

# Reporting Summary

Nature Portfolio wishes to improve the reproducibility of the work that we publish. This form provides structure for consistency and transparency in reporting. For further information on Nature Portfolio policies, see our Editorial Policies and the Editorial Policy Checklist.

## Statistics

For all statistical analyses, confirm that the following items are present in the figure legend, table legend, main text, or Methods section.

| n/a | Confirmed | |
|---|---|---|
| ☐ | ☒ | The exact sample size (*n*) for each experimental group/condition, given as a discrete number and unit of measurement |
| ☐ | ☒ | A statement on whether measurements were taken from distinct samples or whether the same sample was measured repeatedly |
| ☐ | ☒ | The statistical test(s) used AND whether they are one- or two-sided<br>*Only common tests should be described solely by name; describe more complex techniques in the Methods section.* |
| ☒ | ☐ | A description of all covariates tested |
| ☒ | ☐ | A description of any assumptions or corrections, such as tests of normality and adjustment for multiple comparisons |
| ☐ | ☒ | A full description of the statistical parameters including central tendency (e.g. means) or other basic estimates (e.g. regression coefficient) AND variation (e.g. standard deviation) or associated estimates of uncertainty (e.g. confidence intervals) |
| ☐ | ☒ | For null hypothesis testing, the test statistic (e.g. *F*, *t*, *r*) with confidence intervals, effect sizes, degrees of freedom and *P* value noted<br>*Give P values as exact values whenever suitable.* |
| ☒ | ☐ | For Bayesian analysis, information on the choice of priors and Markov chain Monte Carlo settings |
| ☒ | ☐ | For hierarchical and complex designs, identification of the appropriate level for tests and full reporting of outcomes |
| ☒ | ☐ | Estimates of effect sizes (e.g. Cohen's *d*, Pearson's *r*), indicating how they were calculated |

*Our web collection on statistics for biologists contains articles on many of the points above.*

## Software and code

Policy information about availability of computer code

| | |
|---|---|
| Data collection | TECAN infinite M NANO+/F200 Pro/SPARK plate readers were used for OD600 and luminescence measurements. Bio-Rad CFX384 was used for qPCR. |
| Data analysis | Bio-Rad CFX Manager was used to export qPCR data. GraphPad Prism 7 was used to plot RT-qPCR data (bar graphs, line graphs and heat maps), screening data, and survival curves. This software was used to calculate all statistical significance for these assays as described. Java TreeView 1.1.6r4 was used to plot heat maps for growth data. Magellan 7.2 was used to collect data from TECAN plate readers. ZEN pro was used to capture and process fluorescence microscopic images. |

For manuscripts utilizing custom algorithms or software that are central to the research but not yet described in published literature, software must be made available to editors and reviewers. We strongly encourage code deposition in a community repository (e.g. GitHub). See the Nature Portfolio guidelines for submitting code & software for further information.

## Data

Policy information about availability of data

All manuscripts must include a data availability statement. This statement should provide the following information, where applicable:

- Accession codes, unique identifiers, or web links for publicly available datasets
- A description of any restrictions on data availability
- For clinical datasets or third party data, please ensure that the statement adheres to our policy

The datasets generated during and/or analysed during the current study are available from the corresponding author on reasonable request.

## Human research participants

Policy information about studies involving human research participants and Sex and Gender in Research.

| | |
|---|---|
| Reporting on sex and gender | N/A |
| Population characteristics | N/A |
| Recruitment | N/A |
| Ethics oversight | N/A |

Note that full information on the approval of the study protocol must also be provided in the manuscript.

# Field-specific reporting

Please select the one below that is the best fit for your research. If you are not sure, read the appropriate sections before making your selection.

☒ Life sciences ☐ Behavioural & social sciences ☐ Ecological, evolutionary & environmental sciences

For a reference copy of the document with all sections, see nature.com/documents/nr-reporting-summary-flat.pdf

# Life sciences study design

All studies must disclose on these points even when the disclosure is negative.

| | |
|---|---|
| Sample size | Sample size was always n=3 or greater when statistical analysis was required. All experiments were performed in biological duplicate or greater with little deviation between replicates. Selection of these sample sizes was sufficient to observe statistically significant and reproducible results in all experiments. All data is available in Source data file. |
| Data exclusions | No data from successful experiments were excluded. Successful experiments means experiments where both positive and negative controls gave the expected results. |
| Replication | In cases of fungal growth assessment, assays were performed in technical duplicates which were averaged. In all other cases experiments were performed in technical triplicate as indicated in figure legends and methods. Each experiment was performed in at least biological duplicate with both replicates showing similar results. All attempts at replication were successful. |
| Randomization | Randomization was not relevant to the type of experimentation reported. All assays had a quantitative output where statistical analysis was performed, rather than qualitative observations, and therefore, randomization was not required to eliminate user bias. |
| Blinding | Investigators were not blinded when assessing the anti-CD45 IHC data. Three noncontiguous kidney sections from three independent mice were evaluated by multiple (three) investigators independently and the same conclusion was reached. All uncropped images are provided in a supplementary figure. Investigators were not blinded for classical molecular biology and biochemistry experiments as this was not necessary due to the use of statistical analysis to generate conclusions. |

# Reporting for specific materials, systems and methods

We require information from authors about some types of materials, experimental systems and methods used in many studies. Here, indicate whether each material, system or method listed is relevant to your study. If you are not sure if a list item applies to your research, read the appropriate section before selecting a response.

## Materials & experimental systems

| n/a | Involved in the study |
| --- | --- |
| ☐ | ☒ Antibodies |
| ☒ | ☐ Eukaryotic cell lines |
| ☒ | ☐ Palaeontology and archaeology |
| ☐ | ☒ Animals and other organisms |
| ☒ | ☐ Clinical data |
| ☒ | ☐ Dual use research of concern |

## Methods

| n/a | Involved in the study |
| --- | --- |
| ☒ | ☐ ChIP-seq |
| ☒ | ☐ Flow cytometry |
| ☒ | ☐ MRI-based neuroimaging |

# Antibodies

| | |
| --- | --- |
| Antibodies used | anti-Flag: F3165/A8592 (Sigma-Aldrich)<br>anti-HA: sc-7392 or the more concentrated version sc-7392x (Santa Cruz); 3F10 (Roche)<br>anti-CD45: RA3-6B2 (Invitrogen)<br>anti-Candida: ab53891 (Abcam)<br>goat anti-mouse HRP: 1706516 (Bio-Rad)<br>goat anti-rat HRP: A10549 (Invitrogen) |
| Validation | For WB and ChIP, specificity was controlled by including the untagged parental strain in experiments. Staining of mock infected kidneys were used to control specificity of IHC signals by anti-CD45 and anti-Candida. Anti-Flag and anti-HA primary antibodies used in this study have been broadly used for detection of the respective epitope tag with proved specificity (See https://www.sigmaaldrich.com/CA/en/product/sigma/f3165; https://www.scbt.com/p/ha-probe-antibody-f-7; https://www.sigmaaldrich.com/CA/en/product/roche/roahaha for detailed information). The anti-CD45 antibody passed the 'Advanced verification' performed by the vendor. The use of the anti-Candida antibody in IHC is guaranteed by the vendor. Relevant information is available on the vendor websites. |

# Animals and other research organisms

Policy information about studies involving animals; ARRIVE guidelines recommended for reporting animal research, and Sex and Gender in Research

| | |
| --- | --- |
| Laboratory animals | Experiments were performed with 8- to 10-week-old female BALB/c mice (n=8 per experimental group) from Charles River Laboratories (Strain code 028). Animals were provided with fresh water and chow ad libitum. Housing was performed under a 12:12 light: dark cycle in a well-ventilated room (10-15 air changes per hour) with stabilized temperature (68-79 degrees Fahrenheit) and humidity (30-70%). |
| Wild animals | No wild animals were used in this study. |
| Reporting on sex | Female mice were used in the study given their overall smaller size, which has been optimized for the C. albicans inoculum used to establish both systemic and commensal infection. Historically, female mice have been used for C. albicans injections as well due to the fact that female mice are easier to handle and do not fight when caged together, enabling adequate optimization of the inoculum dose (PMID: 35366989). |
| Field-collected samples | No field-collected samples were used in this study. |
| Ethics oversight | All procedures involving mice were approved by the Institutional Animal Care and Use Committee at the University of California San Francisco and were carried out according to the National Institute of Health (NIH) guidelines for the ethical treatment of animals. |

Note that full information on the approval of the study protocol must also be provided in the manuscript.
